# Pamufetinib (TAS-115) for chronic fibrosing interstitial lung diseases with a progressive phenotype: a double-blind, multicenter, phase 2b clinical trial

**DOI:** 10.1093/ajrccm/aamag125

**Published:** 2026-03-22

**Authors:** Ryo Okuda, Yasuhiko Nishioka, Yasuhiro Kondoh, Kazuya Tsubouchi, Masaki Okamoto, Osamu Nishiyama, Seidai Sato, Keiji Oishi, Nobuhisa Ishikawa, Hirofumi Chiba, Yasunari Miyazaki, Sakae Homma, Takashi Ogura, Yoshikazu Inoue, Arata Azuma

**Affiliations:** Department of Respiratory Medicine, Kanagawa Cardiovascular and Respiratory Center, Kanagawa, Japan; Department of Respiratory Medicine and Rheumatology, Graduate School of Biomedical Sciences, Tokushima University, Tokushima, Japan; Department of Respiratory Medicine and Allergy, Tosei General Hospital, Aichi, Japan; Department of Respiratory Medicine and Allergy, Aichi Medical University, Aichi, Japan; Department of Respiratory Medicine, Graduate School of Medical Sciences, Kyushu University, Fukuoka, Japan; Department of Respirology, NHO Kyushu Medical Center, Fukuoka, Japan; Department of Respiratory Medicine and Allergology, Kindai University Faculty of Medicine, Osaka, Japan; Department of Respiratory Medicine and Rheumatology, Graduate School of Biomedical Sciences, Tokushima University, Tokushima, Japan; Department of Respiratory Medicine and Infectious Disease, Graduate School of Medicine, Yamaguchi University, Yamaguchi, Japan; Department of Respiratory Medicine, Hiroshima Prefectural Hospital, Hiroshima, Japan; Department of Respiratory Medicine and Allergology, Sapporo Medical University School of Medicine, Hokkaido, Japan; Department of Respiratory Medicine, Institute of Science Tokyo Hospital, Tokyo, Japan; Department of Respiratory Medicine, Toho University Omori Medical Center, Tokyo, Japan; Department of Respiratory Medicine, Kanagawa Cardiovascular and Respiratory Center, Kanagawa, Japan; Clinical Research Center, NHO Kinki Chuo Chest Medical Center, Osaka, Japan; Department of Internal Medicine, Osaka Anti-Tuberculosis Association Osaka Fukujuji Hospital, Osaka, Japan; Pulmonary Medicine, Tokorozawa Mihara General Hospital, Saitama, Japan; Nippon Medical School, Tokyo, Japan

**Keywords:** antifibrotic agent, idiopathic pulmonary fibrosis, interstitial lung diseases

## Abstract

**Rationale:**

No recommended therapy exists for chronic fibrosing interstitial lung diseases (CF-ILDs) with disease progression despite ongoing treatment with nintedanib or pirfenidone. Pamufetinib (also known as TAS-115) is a novel oral antifibrotic tyrosine kinase inhibitor in development for CF-ILD with a progressive phenotype.

**Objective:**

We sought to evaluate the dose-response of pamufetinib monotherapy in patients with CF-ILD including idiopathic pulmonary fibrosis (IPF) with a progressive phenotype despite an antifibrotic treatment.

**Methods:**

In this double-blind, multicenter, active-controlled phase 2b study, patients with CF-ILD with a progressive phenotype (defined as ≥ 5% decline in the annual percent predicted FVC [%FVC] despite treatment with nintedanib or pirfenidone and an %FVC ≥ 50%) were randomized 1:1:1 to pamufetinib 50 mg, 100 mg, or control (nintedanib or pirfenidone) for ≥ 6 weeks. The primary endpoint was the 26-week rate of decline in FVC.

**Measurements and Main Results:**

Of the 243 patients randomized, approximately 70% had IPF. The 26-week rate of change in FVC was −157.8 mL, −95.9 mL, and −63.6 mL in patients receiving pamufetinib 100 mg, pamufetinib 50 mg, and control, respectively; as such, no clear dose-response relationship was observed. The most frequent adverse event in the pamufetinib groups was rash, which was mostly mild or moderate in severity.

**Conclusions:**

While the safety profile was acceptable, pamufetinib failed to decelerate FVC decline in patients with CF-ILD with a progressive phenotype who had previously been treated with nintedanib or pirfenidone. No benefits were demonstrated by switching from standard antifibrotic treatment to pamufetinib monotherapy.

Clinical trial registered with the Japan Registry of Clinical Trials (https://jrct.mhlw.go.jp/en-top; jRCT2051210050).

At a Glance Commentary **Scientific Knowledge on the Subject:** Recommended treatments are lacking for patients with chronic fibrosing interstitial lung diseases (CF-ILD) including idiopathic pulmonary fibrosis (IPF) with a progressive phenotype despite antifibrotic treatment with nintedanib and/or pirfenidone. The novel oral tyrosine kinase inhibitor pamufetinib delayed disease progression in an exploratory phase 2a study involving patients with IPF (defined as ≥5% decline in annual percent predicted forced vital capacity [%FVC] despite treatment with nintedanib or pirfenidone and %FVC ≥50%) previously treated with nintedanib or pirfenidone. This 26-week, active-controlled phase 2b study evaluated the dose-response of pamufetinib in this population randomized to pamufetinib 50 mg, 100 mg, or control (nintedanib or pirfenidone).

**What This Study Adds to the Field:** Pamufetinib did not appear to decelerate the decline in FVC compared with control after 26 weeks of treatment. This may be partly due to off-target inhibition by pamufetinib, which inhibits multiple kinases, including MET, AXL, and Src, platelet-derived growth factor receptors (PDGFR), vascular endothelial growth factor receptors (VEGFR), and colony stimulating factor-1 receptors (CSF1R). Since MET signaling facilitates repair during lung injury, pamufetinib may impair alveolar epithelial repair, leading to deterioration of respiratory function, acute exacerbations. Switching from standard antifibrotics to pamufetinib did not provide benefits.

## Introduction

Chronic fibrosing interstitial lung diseases (CF-ILDs) with a progressive phenotype, such as idiopathic pulmonary fibrosis (IPF) and progressive pulmonary fibrosis (PPF), are rare diseases with a poor prognosis.^1^ For the treatment of CF-ILD with a progressive phenotype, the antifibrotic agents pirfenidone and nintedanib are approved for IPF, and nintedanib is also approved for PPF. However, there are no currently recommended treatments for patients with CF-ILD with a progressive phenotype that does not respond to antifibrotic therapy with these agents. During the natural course of IPF and PPF, a decrease in FVC of approximately 5% per year is typical,^2-4^ and approximately 40% of patients treated with pirfenidone or nintedanib experience a ≥ 5% decline in FVC per year.^5-7^ Furthermore, there is a significant association between FVC decline and an increased mortality rate.^8^ Therefore, there is an unmet need for a drug that is effective and safe for patients who experience disease progression despite receiving antifibrotic therapy.

Pamufetinib (also known as TAS-115) is an oral tyrosine kinase inhibitor that competes with adenosine triphosphate and exhibits an inhibitory effect on platelet-derived growth factor receptors (PDGFR), vascular endothelial growth factor receptors (VEGFR), and colony-stimulating factor-1 receptors (CSF1R).^9^ Its highly potent PDGFR inhibition blocks the proliferation and migration of fibroblasts, and its CSF1R inhibition suppresses the macrophage activation that promotes fibrosis. In a phase 2a single-arm study of pamufetinib, which enrolled treatment-naive patients with IPF as well as patients who experienced a decline in FVC despite receiving pirfenidone or nintedanib treatment,^10^ pamufetinib was effective in delaying progression of IPF, assessed using the intrapatient change in the slope of %FVC decline as a surrogate endpoint.^11^ The aim of the current study was to evaluate the dose response of pamufetinib in patients with CF-ILD including IPF with a progressive phenotype who had been treated with pirfenidone and/or nintedanib. Some of the results of this study have been previously reported in the form of an abstract at the American Thoracic Society 2025 International Congress.^12^

## Methods

### Study design

This double-blind, parallel-group, phase 2b study was conducted at 41 sites in Japan between October 2021 and June 2024 (Japan Registry of Clinical Trials identifier: jRCT2051210050). Patients were randomized 1:1:1 to pamufetinib 100 mg, pamufetinib 50 mg, or control (nintedanib or pirfenidone) for 26 weeks. Patients in the control group continued to receive the antifibrotic agent they had received prior to randomization. See the online supplement for further study design details (Figure S1).

**Figure 1:**
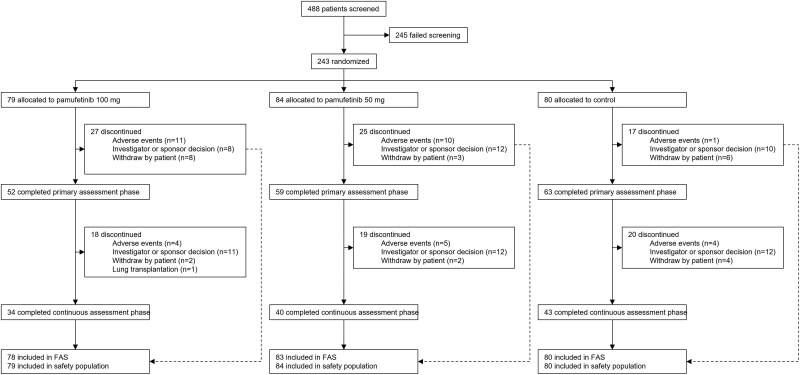
Patient disposition. Abbreviation: FAS, full analysis set.

This study was conducted in accordance with the Declaration of Helsinki and Good Clinical Practice Guidelines. The study protocol and informed consent forms were approved by the institutional review board at each study site. All patients provided written informed consent before the initiation of any study-specific procedures.

### Participants

Adults (aged 20 years and older) with ILD who had received nintedanib or pirfenidone for ≥ 90 days prior to screening were enrolled. Patients were required to have (1) fibrosis in > 10% of the total lung field, confirmed by high-resolution computed tomography; (2) a relative %FVC decline ≥ 5% per year; (3) an %FVC ≥ 50%; (4) a percent predicted DLco (%DLco) ≥ 25%, corrected for hemoglobin; and (5) greater fibrosis extent than emphysematous lesions. Patients with a prebronchodilator FEV_1_/FVC ratio < 0.7, a diagnosis of severe pulmonary hypertension, or a history of acute exacerbation of ILD were excluded. See the online supplement for the full study inclusion and exclusion criteria.

### Randomization and masking

Once the eligibility of the study participants had been confirmed and the criteria for randomization were met, investigators randomized the patients using a central randomization using an interactive web response system (IWRS). Patients were randomized in a 1:1:1 ratio to pamufetinib 100 mg, pamufetinib 50 mg, or control treatment (nintedanib or pirfenidone) via the IWRS based on a minimization method. Allocation adjustment factors were prior therapy (nintedanib or pirfenidone) and decreased %FVC (rate of ≥ 5% per year and < 10% vs ≥ 10% per year).

Blinding was maintained using the double-dummy method combining the active drugs and matching placebos because the dosage forms and regimens of pamufetinib, nintedanib, and pirfenidone were different. Pamufetinib was administered orally on a 5-day on/2-day off schedule. Nintedanib or pirfenidone were administered daily. Placebos of each drug were utilized to maintain blinding. The patients, medical staff, investigators, and sponsors were blinded to the treatment assignments. Blinding was maintained even after treatment.

### Outcomes

The primary endpoint was the 26-week rate of decline in FVC. Forced vital capacity was measured using sponsor-provided spirometers and was centrally reviewed. Key secondary endpoints were absolute change from baseline in FVC, %FVC, and %DLco; time to first acute exacerbation; and overall survival. Safety was assessed using the Common Terminology Criteria for Adverse Events, version 5.0, and the Medical Dictionary for Regulatory Activities, version 27.0. Quality of life was assessed using the St. George’s Respiratory Questionnaire (SGRQ), with response defined as an absolute score change of −4 points.

### Statistical analysis

Based on previous studies,^11^^,^^13^^,^^14^ it was assumed that the mean (SD) difference in the 26-week rate of decline in FVC between the pamufetinib and control groups would be 100 mL (200 mL). Using the Monte Carlo simulation (1-sided significance level 2.5%), we estimated that 72 patients per treatment group would be needed to achieve 90% statistical power for comparing dose-response relationships. Assuming 10% of patients had missing data, a sample size of 80 per group (240 patients total) was set.

Safety was assessed in the safety population, which comprised all patients who received ≥ 1 dose of pamufetinib, pirfenidone, or nintedanib in the study. Efficacy was assessed in the full analysis set, which included all patients in the safety population who had ≥ 1 postrandomization FVC measurement.

Descriptive statistics were used to summarize the baseline characteristics and adverse events (AEs), with means and SDs used for continuous variables and number and proportions of patients used for categorical variables. See the online supplement for the statistical methodology used to conduct the primary analysis.

## Results

### Patient characteristics

From October 2021 to November 2023, a total of 243 patients underwent randomization and received at least 1 dose of either pamufetinib or control drugs (nintedanib or pirfenidone) (79 in the pamufetinib 100 mg group, 84 in the pamufetinib 50 mg group, and 80 in the control group). The demographic and baseline characteristics are summarized in Table 1 and Tables S1 and S2 in the online supplement. The patient characteristics were generally well balanced between treatment groups. The characteristics of the overall patient population were similar to those of each individual group (Table 1). Most patients were male (84.2%), and the mean age was 70.2 (±8.5) years in overall patients. The most frequent diagnoses of ILD were IPF in 71.0% of patients, followed by unclassifiable idiopathic interstitial pneumonia in 11.2% of patients (Table S1 in the online supplement). The mean value of %FVC was almost the same across each treatment group (73.78 ± 15.38 in the pamufetinib 100 mg group; 73.99 ± 12.86 in the pamufetinib 50 mg group; 73.99 ± 14.60 in the control group). The mean treatment compliance rate up to 26 weeks exceeded 95% in every treatment group. The completion rate of 26-week administration was highest in the control group, and although slight, it decreased with increasing doses of pamufetinib in the pamufetinib group experienced a higher frequency of discontinuation due to AEs, regardless of the dose administered (Figure 1).

**Table 1:** Baseline characteristics in the full analysis set.

	**Pamufetinib 100 mg (*n*** = **78)**	**Pamufetinib 50 mg (*n*** = **83)**	**Control (*n*** = **80)**
**Male sex, *n* (%)**	71 (91.0)	68 (81.9)	64 (80.0)
**Age, years, mean ± SD**	69.0 ± 9.6	70.3 ± 7.5	71.2 ± 8.3
**Former or current smoker, *n* (%)**	63 (80.8)	65 (78.3)	64 (80.0)
**IPF, *n* (%)**	57 (73.1)	61 (73.5)	53 (66.3)
**Duration of ILD from initial diagnosis, mean ± SD, years**	4.0 ± 2.7	4.8 ± 3.3	3.8 ± 2.7
**Duration of prior nintedanib and/or pirfenidone, mean ± SD, years**	2.1 ± 1.7	1.9 ± 1.5	2.0 ± 1.4
**Prior antifibrotic treatment, *n* (%)**			
** Nintedanib**	57 (73.1)	64 (77.1)	60 (75.0)
** Pirfenidone**	14 (17.9)	12 (14.5)	12 (15.0)
** Both**	7 (9.0)	7 (8.4)	8 (10.0)
**FVC, mean ± SD**			
** Value, mL**	2397.2 ± 557.9	2263.7 ± 531.7	2238.5 ± 569.2
** % predicted**	73.8 ± 15.4	74.0 ± 12.9	74.0 ± 14.6
**Decline in %FVC, *n* (%)**			
** ≥5% and < 10% per year**	39 (50.0)	42 (50.6)	41 (51.3)
** ≥10% per year**	39 (50.0)	41 (49.4)	39 (48.8)
**DLco, mean ± SD**			
** Value, mL/min/mmHg,**	9.0 ± 3.0	9.3 ± 3.2	8.4 ± 2.9
** % predicted**	53.5 ± 15.2	57.0 ± 17.7	52.5 ± 16.8

Abbreviations: %FVC, percent predicted FVC; ILD, interstitial lung disease; IPF, idiopathic pulmonary fibrosis.

**Table 2:** Primary endpoint and main secondary lung-function endpoints in the full analysis set.

	**Pamufetinib 100 mg (*n*** = **78)**	**Pamufetinib 50 mg (*n*** = **83)**	**Control (*n*** = **80)**
**Primary endpoint**			
**26-week rate of decline in FVC, mL, mean (95% CI)**			
** Overall population**	−157.8 (−208.8, −106.9)	−96.0 (−145.4, −46.6)	−63.6 (−111.8, −15.5)
** IPF patients** ^a^	−100.7 (−163.0, −38.3)	−85.6 (−145.0, −26.2)	−64.0 (−125.6, −2.4)
** Non-IPF patients** ^b^	−350.6 (−447.8, −253.5)	−154.0 (−257.9, −50.2)	−107.3 (−197.0, −17.5)
**Key secondary endpoints**			
**26-week absolute %DLco change from baseline, % (95% CI)**			
** Overall population**	−5.2 (−8.7, −1.8)	−3.4 (−6.7, −0.0)	−2.6 (−5.9, 0.7)
** IPF patients** ^a^	−5.3 (−8.7, −1.8)	−3.6 (−6.9, −0.4)	−5.9 (−9.4, −2.4)
** Non-IPF patients** ^b^	−5.0 (−14.1, 4.0)	−2.5 (−11.6, 6.7)	4.0 (−3.5, 11.4)
**Acute exacerbation at week 26, *n* (%)**			
** Overall population**	10 (12.8)	8 (9.6)	3 (3.8)
** IPF patients** ^a^	5 (8.8)	6 (9.8)	2 (3.8)
** Non-IPF patients** ^b^	5 (23.8)	2 (9.1)	1 (3.7)
**Death at week 26, *n* (%)**			
** Overall population**	3 (3.8)	5 (6.0)	2 (2.5)
** IPF patients** ^a^	1 (1.8)	4 (6.6)	1 (1.9)
** Non-IPF patients** ^b^	2 (9.5)	1 (4.5)	1 (3.7)

Abbreviations: ILD, interstitial lung disease; IPF, idiopathic pulmonary fibrosis.

a *n* = 57 (pamufetinib 100 mg), 61 (pamufetinib 50 mg), and 53 (control).

b *n* = 21 (pamufetinib 100 mg), 22 (pamufetinib 50 mg), and 27 (control).

### Efficacy

**Figure 2:**
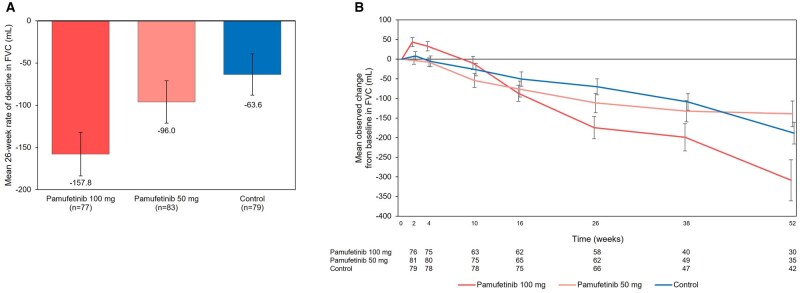
Forced vital capacity in the full analysis set (A) 26-week rate of decline and (B) change from baseline over time. Error bars indicate standard error. For Figure 2A, a linear mixed effect model was conducted, with treatment group, baseline value, degree of emphysema, assessment time, interaction between treatment group included as random effects, and interaction between baseline value and assessment time as fixed effects. Abbreviation: FVC, forced vital capacity.

The 26-week rate of change in FVC was estimated to be −157.8 mL (95% CI, −208.8 to −106.9), −96.0 mL (95% CI, −145.4 to −46.6), and −63.6 mL (95% CI, −111.8 to −15.5) in the pamufetinib 100 mg, pamufetinib 50 mg, and control group, respectively (Figure 2A). Although the high-dose descending type was selected as the dose response pattern, it was not statistically significant (see Table S3 in the online supplement). In the pamufetinib 100 mg group, while the FVC decline was suppressed until week 10, it eventually became greater than the decline in the control group by week 26 (Figure 2B). An analysis of FVC decline based on the presence or absence of skin disorder related to study drug in patients with IPF revealed that in patients who experienced a skin disorder, pamufetinib 100 mg tended to decelerate the FVC decline compared with the control group (Figure S2 in the online supplement).

**Table 3:** Adverse events up to week 26 in the safety population.

	**Pamufetinib 100 mg *n* (%)^a^ (*n*** = **79)**	**Pamufetinib 50 mg *n* (%)^a^ (*n*** = **84)**	**Control *n* (%)^a^ (*n*** = **80)**
**Any AE**	73 (92.4)	71 (84.5)	71 (88.8)
**Most frequent AEs^b^**			
** Rash**	23 (29.1)	12 (14.3)	8 (10.0)
** Diarrhea**	5 (6.3)	4 (4.8)	16 (20.0)
** Pyrexia**	8 (10.1)	5 (6.0)	8 (10.0)
** COVID-19**	5 (6.3)	9 (10.7)	7 (8.8)
** ILD^c^**	8 (10.1)	8 (9.5)	3 (3.8)
** Constipation**	8 (10.1)	5 (6.0)	3 (3.8)
** Decreased appetite**	2 (2.5)	1 (1.2)	11 (13.8)
**Severe AEs^d^**	20 (25.3)	15 (17.9)	11 (13.8)
**Serious AEs^d^**	22 (27.8)	17 (20.2)	12 (15.0)
**Fatal AEs**	3 (3.8)	5 (6.0)	2 (2.5)
**AEs leading to treatment discontinuation**	10 (12.7)	10 (11.9)	0

Abbreviations: AE, adverse event; CTCAE, Common Terminology Criteria for Adverse Events; ILD, interstitial lung disease; MedDRA, Medical Dictionary for Regulatory Activities.

a Adverse events were coded using MedDRA, version 27.0.

b The most frequent AEs were defined as those with an incidence > 10% in any study group.

c Interstitial lung disease was reported as an AE, encompassing both acute ILD exacerbations and newly diagnosed ILD.

d A severe AE was defined as an AE of grade ≥ 3 in the CTCAE, version 5.0. A serious AE was defined as any AE that resulted in death, was life-threatening, required inpatient hospitalization or prolongation of existing hospitalization to treat the AE, resulted in persistent or significant disability/incapacity, was a congenital anomaly/birth defect, or was deemed serious for any other reason.

The changes in the %DLco were −5.2, −3.4, and −2.6 in the pamufetinib 100 mg, pamufetinib 50 mg, and control group, respectively. Acute exacerbation of ILD was reported in every group, and pamufetinib 100 mg showed the highest rate of 12.8% among all groups (pamufetinib 50 mg, 9.6% mg; control group, 3.8%; Table 2;  Table S4 and Figure S3 gure S3 in the online supplement). Due to the very limited number of death events, there were no notable differences in overall survival at week 26 among the groups (pamufetinib 100 mg, 3 events; pamufetinib 50 mg, 5 events; control, 2 events; see Table S5 and Figure S4 in the online supplement).

### Safety

Adverse events that occurred up to week 26 are shown in Table 3 and Tables S6, S7, and S8 in the online supplement. The most frequent AE according to the preferred term in the pamufetinib 100 mg group was rash, and this was the same in the pamufetinib 50 mg group. Most of rashes reported as AEs were of mild or moderate severity. The most frequent AE in the control group was diarrhea. In all treatment groups, ILD was reported as an AE, encompassing both acute exacerbations of ILD and newly diagnosed ILD. The incidence rate of ILD was 10.1% in the pamufetinib 100 mg group and 3.8% in the control group, showing a considerable difference. COVID-19 was observed in more than 5% of patients in each of the 3 groups, and the incidence rate was almost the same across all groups. A total of 51 serious AEs were reported, but there was no significant difference in the rate of serious AEs in the pamufetinib 100 mg (27.8%), pamufetinib 50 mg (20.2%), and control groups (15.0%; Table 3). Rashes in the pamufetinib 100 mg group and ILD in the pamufetinib 50 mg group were the common AEs leading to drug discontinuation (≥2 patients), while no such AEs were observed in the control group. Adverse events leading to death occurred in 3.8% (*n* = 3) patients in pamufetinib 100 mg, 6.0% (*n* = 5) patients in pamufetinib 50 mg, and 2.5% (*n* = 2) ­patients in the control group, with these findings indicating that the rates were almost the same across the 3 groups (Table S9 in the online supplement).

### Patient-reported outcome

An absolute change in SGRQ questionnaire total, below −4 points was defined as a responder in the protocol, and the percentage of responders at weeks 26 and 52 were almost the same across the 3 treatment groups (Figure S5 in the online supplement).

## Discussion

In this study, neither pamufetinib 100 mg nor 50 mg improved the rate of decline in FVC compared with the control group after 26 weeks of treatment. The FVC decline in the pamufetinib 100 mg group was suppressed until week 10, which was consistent with the previous phase 2a study.^11^ However, after week 16, the rate of FVC decline was reversed, resulting in an FVC that was lower in the pamufetinib 100 mg group than the control group. Moreover, the pamufetinib 100 mg group exhibited a greater rate of decline in FVC than the pamufetinib 50 mg group. Additionally, the incidence of acute exacerbation was higher in the pamufetinib 100 mg and pamufetinib 50 mg groups compared with the control group, with a dose-dependent increase observed, being more frequent in the 100 mg group.

Regarding the study design, it is possible that discontinuing available antifibrotic therapy in the active treatment arm and switching to pamufetinib monotherapy may be associated with detrimental outcomes. However, in the phase 2a study, after patients with IPF discontinued treatment with the approved antifibrotic agents nintedanib or pirfenidone, which had failed to suppress the decline in FVC, pamufetinib monotherapy effectively delayed disease progression.^11^ At 3 months, there was no negative tendency after discontinuing the approved antifibrotic agents and switching to pamufetinib administration in the phase 2a study. In addition, it has been reported that switching to a second antifibrotic agent can significantly improve survival.^15^ Pamufetinib, similar to nintedanib, shows antifibrotic effects through PDGFR inhibition. These suggest that the unexpected clinical course during pamufetinib treatment observed in this study may have been due to off-target actions of pamufetinib rather than the discontinuation of approved antifibrotic agents.

Pamufetinib inhibits multiple kinases, such as MET, AXL, and Src, in addition to PDGFR, VEGFR, and CSF1R.^16^ Among them, MET is reported to have a pro-repair effect during lung injury.^17^ It is possible that pamufetinib induced the deterioration of respiratory function, including acute exacerbations, by impairing the repair function of alveolar epithelial damage due to MET inhibition. Since FVC decreases following an acute exacerbation, regardless of fibrosis progression, onset of acute exacerbation may have influenced the FVC decline in the pamufetinib groups. Although it was reported that FVC change over 3 months may hold potential as a surrogate endpoint in IPF adaptive trials,^18^ the present study may sound an alarm about the evaluation in short-term treatment. However, no notable difference in the risk of death was observed in either group, although the impact of reduced FVC on overall survival may not be fully evaluated within the limited period of 26 weeks.

The results of the subgroup analyses stratified by IPF and non-IPF status, as well as by prior antifibrotic treatment, were consistent with the overall results, indicating no improvement in FVC with pamufetinib groups compared with the control group. Furthermore, although the sample size was limited, the difference in the 26-week rate of decline in FVC between the control group and the pamufetinib 100 mg group was greater in the non-IPF subgroup than in the IPF subgroup. This could be attributed to the generally higher levels of inflammatory mediators found in non-IPF patients,^19^ indicating a possible pamufetinib-related exacerbation of inflammation in this subgroup. The mechanism likely involves CSF1R inhibition by pamufetinib, leading to sustained peripheral blood monocyte depletion and negatively impacting the homeostasis of immunoregulatory macrophages required for resolving inflammation. Consequently, the elevation in macrophage colony-stimulating factor levels due to monocyte depletion might have aggravated the ongoing inflammatory response.^20^

In the IPF subgroup, it is suggested that patients who developed skin disorders may have exhibited a potential efficacy in decelerating FVC decline, indicating the possibility of high responsiveness to the drug. However, the underlying mechanism of the drug-induced skin disorders remains undetermined.

The safety profile was consistent with the previous phase 2a study.^11^ Compared with the previous phase 2a study, the incidence of AEs tended to be lower in this phase 2b study due to the reduced pamufetinib dose. The number of patients who discontinued treatment due to AEs in this study was higher in the pamufetinib group than in the control group. This is likely because the control group continued their pre-enrollment treatment without experiencing severe new AEs during the study. Although direct comparison is difficult due to different patient backgrounds and assessment periods, the discontinuation rates were 25.2%, 23.7%, and 19.6% for nintedanib in the INPULSIS-1, INPULSIS-2, and INBUILD studies, respectively,^4^^,^^21^^,^^22^ and 19.8% for pirfenidone in the ASCEND study.^23^ The discontinuation rate for pamufetinib in this study was approximately 17% in the pamufetinib 100 mg and pamufetinib 50 mg groups, which is considered acceptable.

There are several limitations to the interpretation of the study results. First, this study included patients with worsened respiratory function (%FVC decreased by ≥ 5%/year) despite the use of antifibrotic agents. Therefore, it is unknown whether the use of pamufetinib can be generalized to untreated patients and to those with %FVC decreased by less than 5%/year. Also, the small number of non-IPF patients necessitates cautious interpretation of the results for this population. Furthermore, this study was conducted only on Japanese patients; therefore, it is unclear whether these data can be generalized to the global population.

## Conclusions

In conclusion, while the safety profile was acceptable, pamufetinib failed to decelerate the decline in FVC among patients with CF-ILD with a progressive phenotype who had previously been treated with nintedanib or pirfenidone. No dose-response relationship was observed for pamufetinib, and no benefits were demonstrated by switching to pamufetinib monotherapy.

## Supplementary Material

aamag125_Supplementary_Data

## Data Availability

Anonymized, patient-level, analyzable datasets will not be shared according to the sponsor policy on data sharing (https://www.taiho.co.jp/en/science/policy/clinical_trial_information_disclosure_policy/index.html).
