# Supplementary material for: Pamufetinib (TAS-115) for chronic fibrosing interstitial lung diseases with a progressive phenotype: a double-blind, multicenter, phase 2b clinical trial
Source: Am J Respir Crit Care Med. 2026 Mar 22;212(8):1770–7. doi: 10.1093/ajrccm/aamag125 (PMC13424673; doi:10.1093/ajrccm/aamag125)
Supplement: aamag125_Supplementary_Data [file aamag125_supplementary_data.zip › Okuda_et_al_editable_figures_supp.pdf]

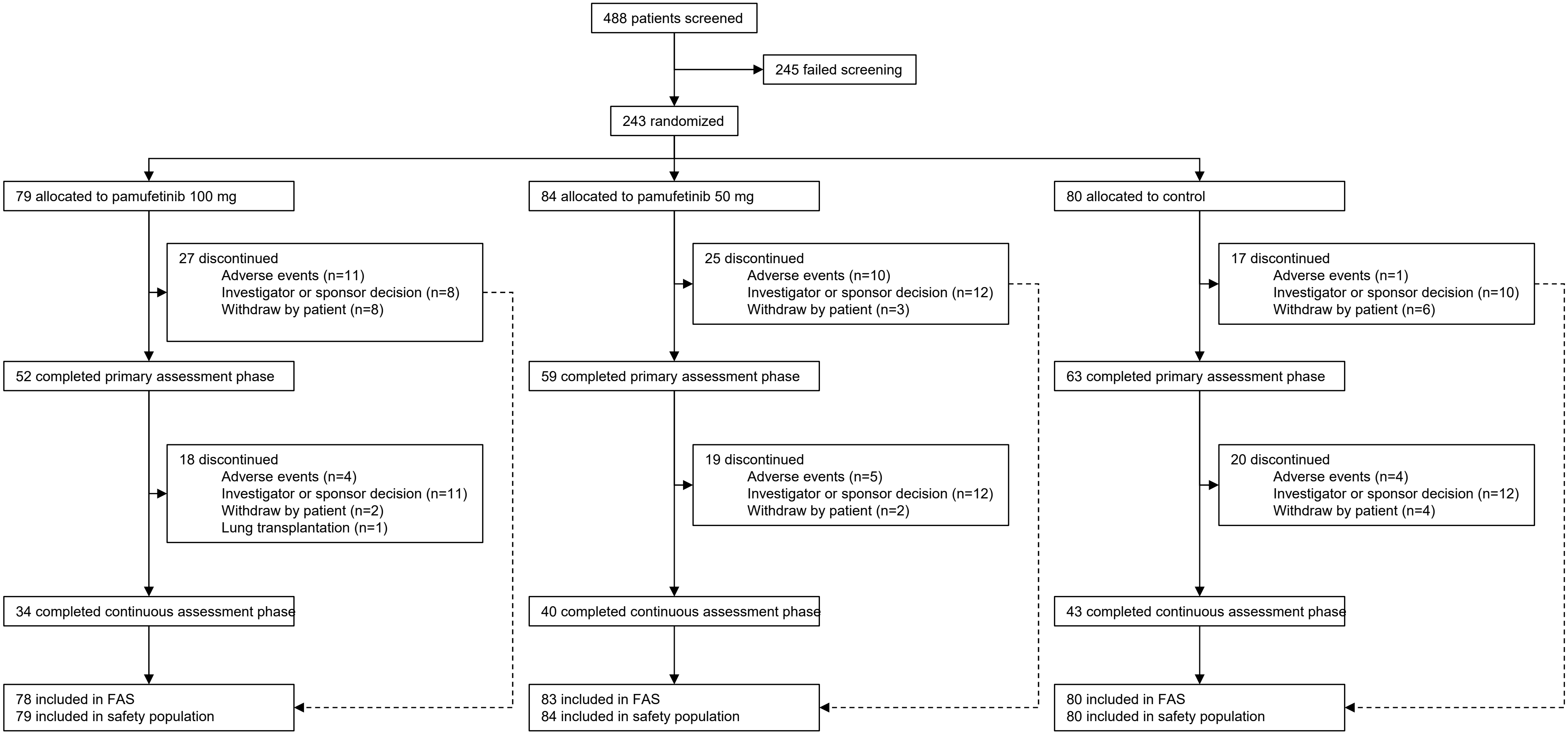
488 patients screened
245 failed screening
243 randomized
79 allocated to pamufetinib 100 mg
84 allocated to pamufetinib 50 mg
80 allocated to control
27 discontinued
Adverse events (n=11)
Investigator or sponsor decision (n=8)
Withdraw by patient (n=8)
25 discontinued
Adverse events (n=10)
Investigator or sponsor decision (n=12)
Withdraw by patient (n=3)
17 discontinued
Adverse events (n=1)
Investigator or sponsor decision (n=10)
Withdraw by patient (n=6)
52 completed primary assessment phase
59 completed primary assessment phase
63 completed primary assessment phase
18 discontinued
Adverse events (n=4)
Investigator or sponsor decision (n=11)
Withdraw by patient (n=2)
Lung transplantation (n=1)
19 discontinued
Adverse events (n=5)
Investigator or sponsor decision (n=12)
Withdraw by patient (n=2)
20 discontinued
Adverse events (n=4)
Investigator or sponsor decision (n=12)
Withdraw by patient (n=4)
34 completed continuous assessment phase
40 completed continuous assessment phase
43 completed continuous assessment phase
78 included in FAS
79 included in safety population
83 included in FAS
84 included in safety population
80 included in FAS
80 included in safety population

(a)

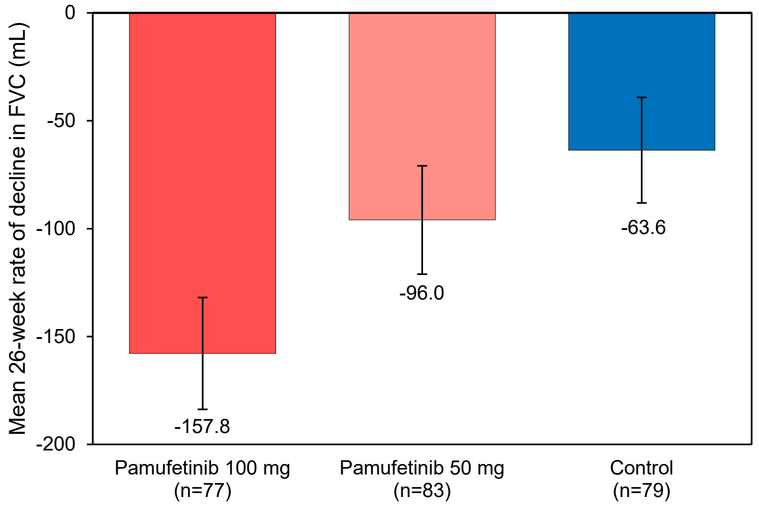

0
-50
-100
-150
-200
Mean 26-week rate of decline in FVC (mL)
-157.8
-96.0
-63.6
Pamufetinib 100 mg
(n=77)
Pamufetinib 50 mg
(n=83)
Control
(n=79)


(b)

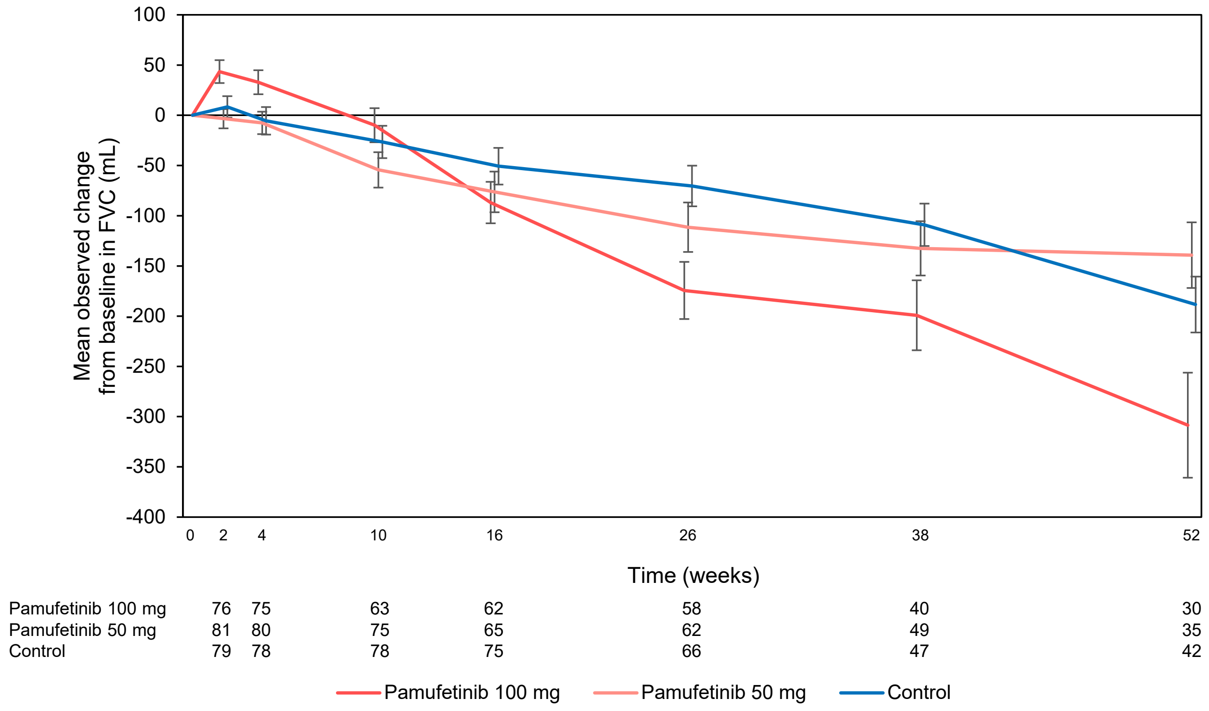

100
50
0
-50
-100
-150
-200
-250
-300
-350
-400
Mean observed change from baseline in FVC (mL)
0 2 4 10 16 26 38 52
Time (weeks)
Pamufetinib 100 mg 76 75 63 62 58 40 30
Pamufetinib 50 mg 81 80 75 65 62 49 35
Control 79 78 78 75 66 47 42
Pamufetinib 100 mg
Pamufetinib 50 mg
Control

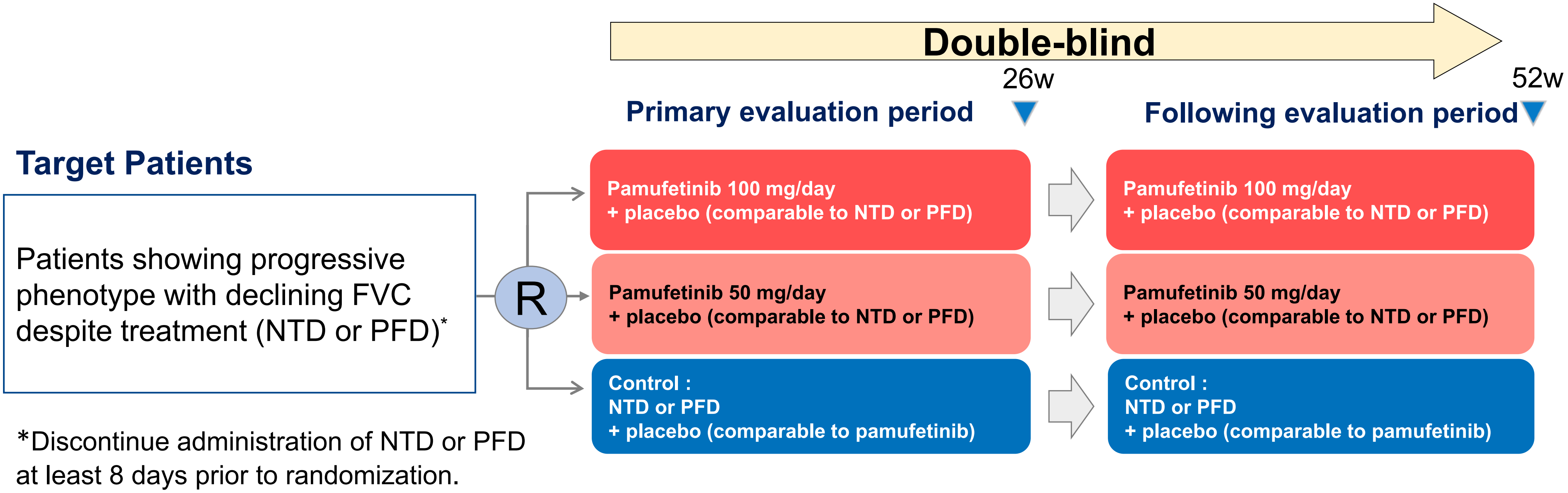

Double-blind
26w
52w
Primary evaluation period
Following evaluation period
Target Patients
Patients showing progressive phenotype with declining FVC despite treatment (NTD or PFD)*
R
Pamufetinib 100 mg/day
+ placebo (comparable to NTD or PFD)
Pamufetinib 50 mg/day
+ placebo (comparable to NTD or PFD)
Control :
NTD or PFD
+ placebo (comparable to pamufetinib)
Pamufetinib 100 mg/day
+ placebo (comparable to NTD or PFD)
Pamufetinib 50 mg/day
+ placebo (comparable to NTD or PFD)
Control :
NTD or PFD
+ placebo (comparable to pamufetinib)
*Discontinue administration of NTD or PFD at least 8 days prior to randomization.

(a) IPF Population

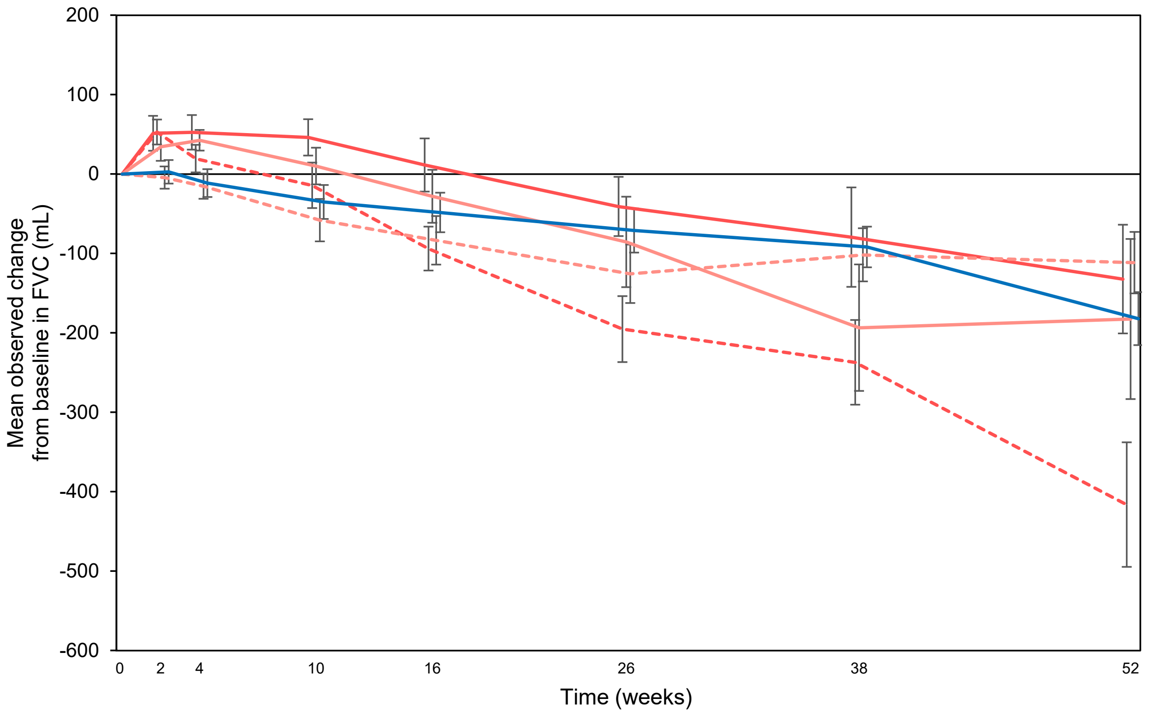


| | 2 | 4 | 10 | 16 | 26 | 38 | 52 |
|---|---|---|---|---|---|---|---|
| Pamufetinib 100 mg with skin disorder | 22 | 23 | 18 | 19 | 17 | 11 | 10 |
| Pamufetinib 100 mg without skin disorder | 33 | 33 | 28 | 27 | 26 | 18 | 15 |
| Pamufetinib 50 mg with skin disorder | 17 | 17 | 17 | 15 | 12 | 10 | 8 |
| Pamufetinib 50 mg without skin disorder | 43 | 42 | 39 | 35 | 36 | 29 | 20 |
| Control | 53 | 52 | 52 | 50 | 43 | 31 | 28 |

Pamufetinib 100 mg with skin disorder
Pamufetinib 100 mg without skin disorder
Pamufetinib 50 mg with skin disorder
Pamufetinib 50 mg without skin disorder
Control

(b) Non-IPF Population

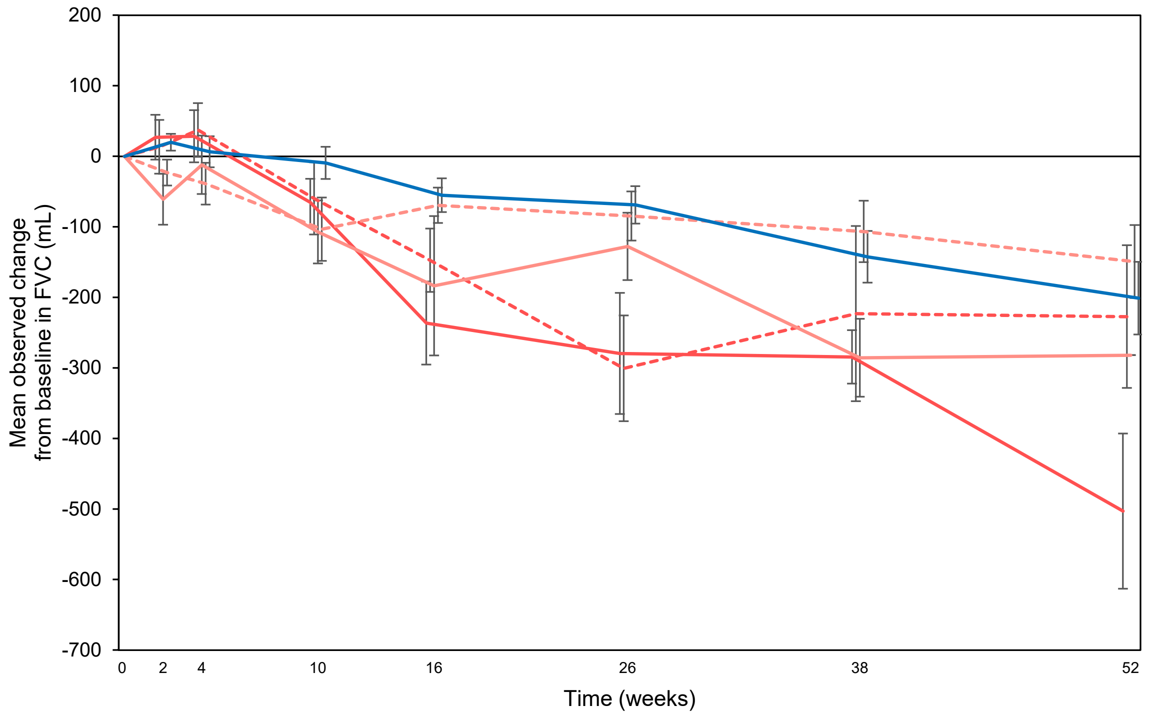


| | | | | | | | |
|---|---|---|---|---|---|---|---|
| Pamufetinib 100 mg with skin disorder | 11 | 10 | 8 | 8 | 8 | 6 | 2 |
| Pamufetinib 100 mg without skin disorder | 10 | 9 | 9 | 8 | 7 | 5 | 3 |
| Pamufetinib 50 mg with skin disorder | 5 | 5 | 5 | 5 | 4 | 3 | 1 |
| Pamufetinib 50 mg without skin disorder | 16 | 16 | 14 | 10 | 10 | 7 | 6 |
| Control | 26 | 26 | 26 | 25 | 23 | 16 | 14 |

(a) Overall Population

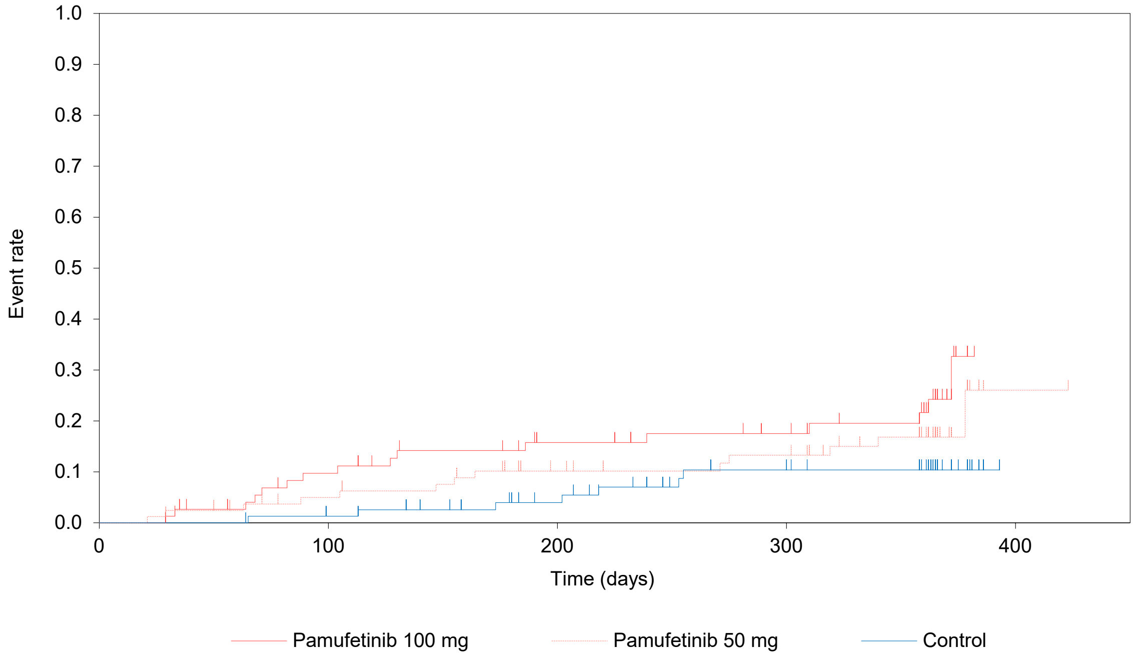

(b) IPF Population

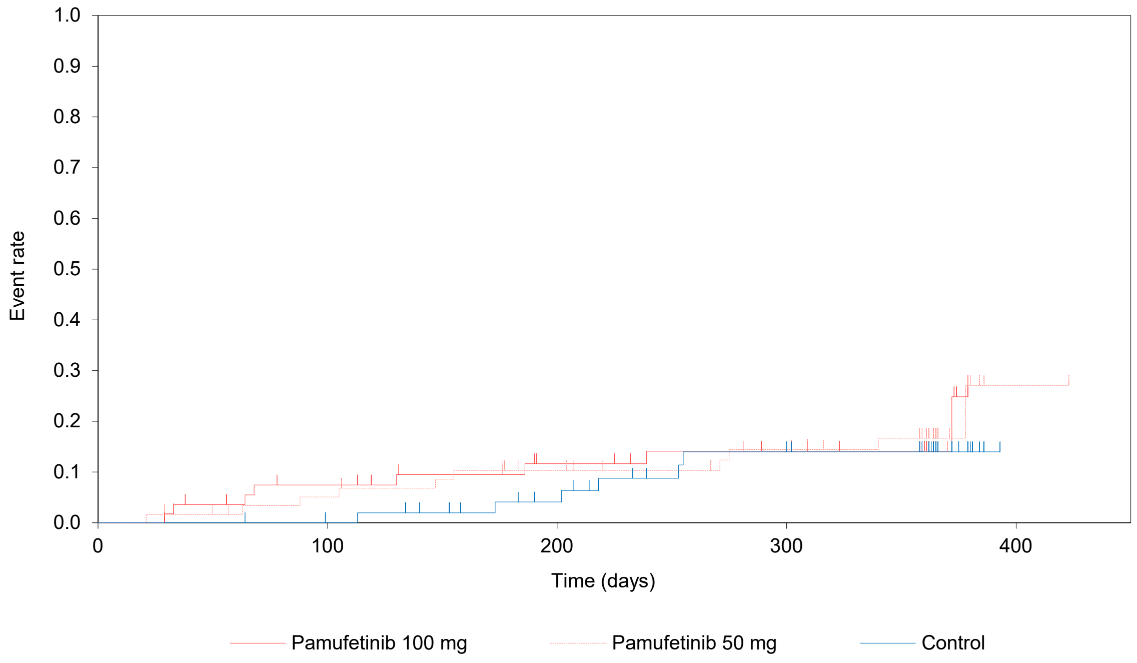

(c) Non-IPF Population

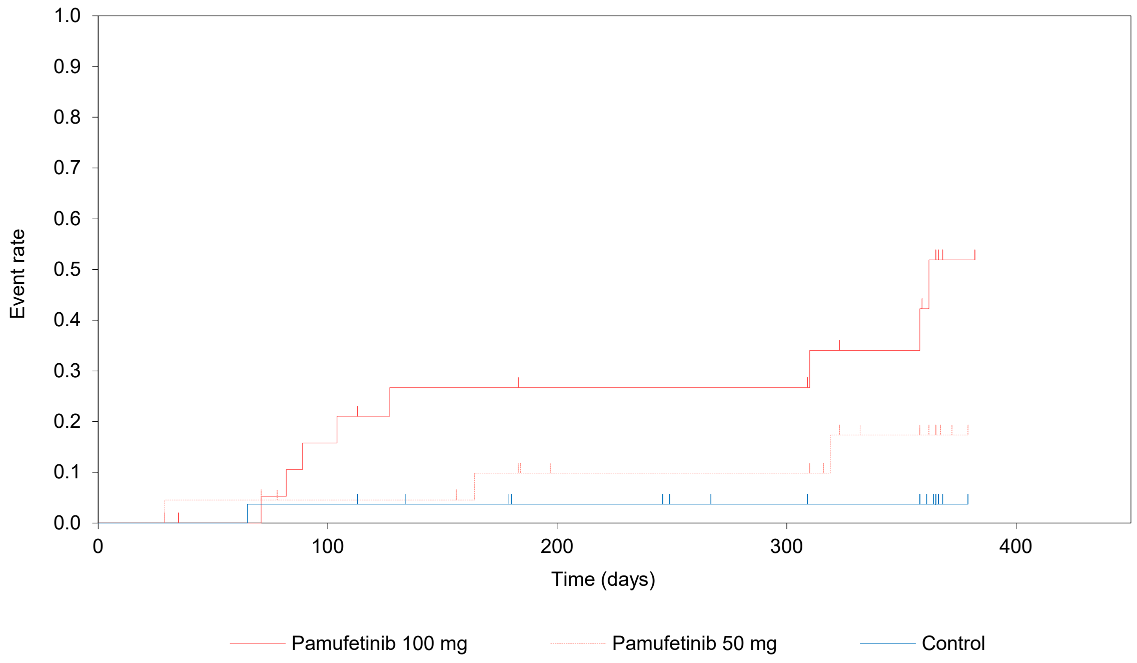

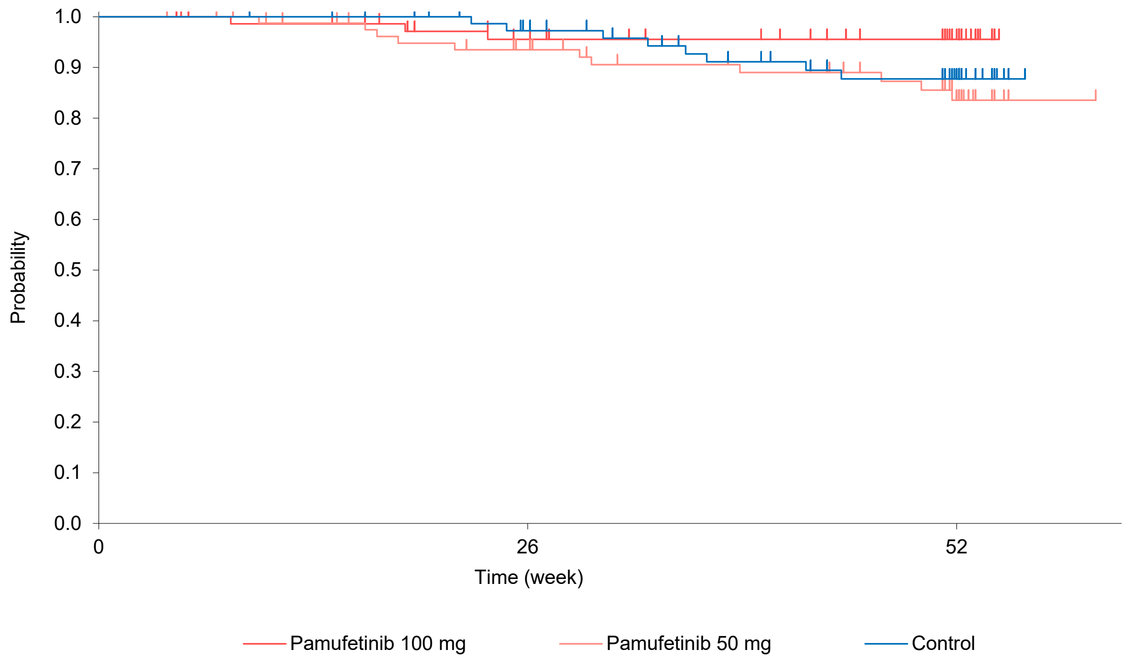

1.0
0.9
0.8
0.7
0.6
0.5
0.4
0.3
0.2
0.1
0.0
Probability
0
26
52
Time (week)
Pamufetinib 100 mg
Pamufetinib 50 mg
Control

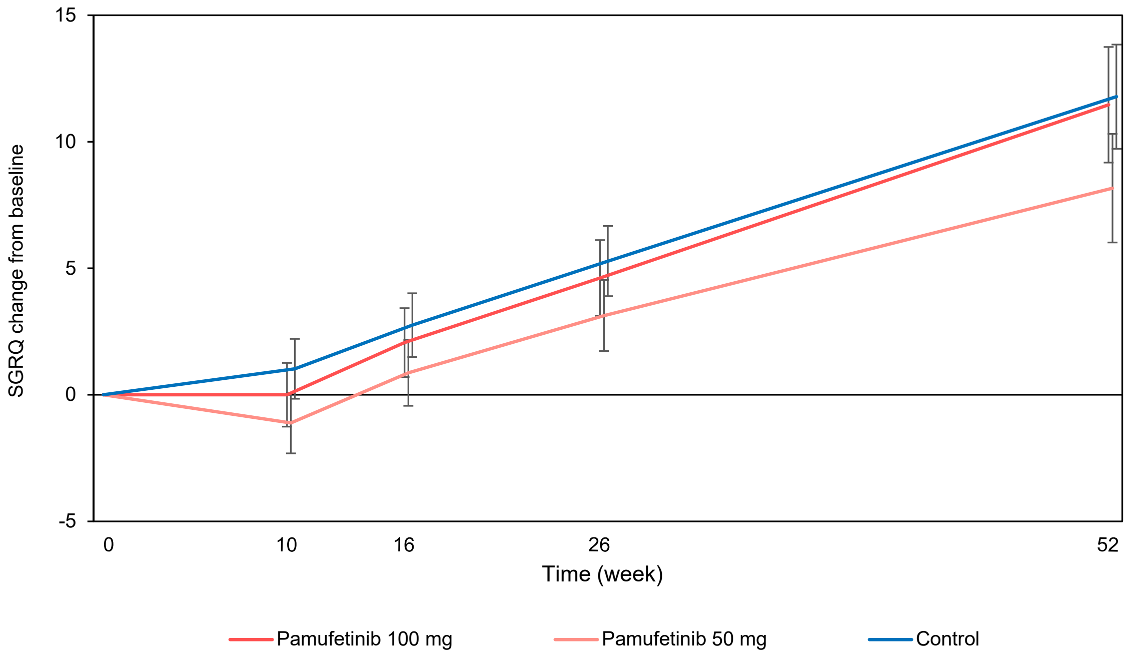

15
10
5
0
-5
SGRQ change from baseline
0
10
16
26
52
Time (week)
Pamufetinib 100 mg
Pamufetinib 50 mg
Control
