# Supplementary material for: Pamufetinib (TAS-115) for chronic fibrosing interstitial lung diseases with a progressive phenotype: a double-blind, multicenter, phase 2b clinical trial
Source: Am J Respir Crit Care Med. 2026 Mar 22;212(8):1770–7. doi: 10.1093/ajrccm/aamag125 (PMC13424673; doi:10.1093/ajrccm/aamag125)
Supplement: aamag125_Supplementary_Data [file aamag125_supplementary_data.zip › Okuda_et_al_online_supplement.docx]

**Pamufetinib (TAS-115) for Chronic Fibrosing Interstitial Lung Diseases with a Progressive Phenotype: A double-blind, multicenter, phase 2b Clinical Trial**

Ryo Okuda, Yasuhiko Nishioka, Yasuhiro Kondoh, Kazuya Tsubouchi, Masaki Okamoto, Osamu Nishiyama, Seidai Sato, Keiji Oishi, Nobuhisa Ishikawa, Hirofumi Chiba, Yasunari Miyazaki, Sakae Homma, Takashi Ogura, Yoshikazu Inoue, Arata Azuma

**Online Supplement**

List of Investigators

| **Site Name** | **Investigator Name** |
| --- | --- |
| Institute of Science Tokyo Hospital | Yasunari Miyazaki |
| Center Hospital of the National Center for Global Health and Medicine | Shinyu Izumi |
| Kinki-Chuo Chest Medical Center | Toru Arai |
| Nagasaki University Hospital | Noriho Sakamoto |
| Tosei General Hospital | Yasuhiro Kondoh |
| Hamamatsu University Hospital | Takafumi Suda |
| National Hospital Organization Kyushu Medical Center | Masaki Okamoto |
| National Hospital Organization Kochi National Hospital | Eiji Takeuchi |
| Kanagawa Cardiovascular and Respiratory Center | Ryo Okuda |
| JR Tokyo General Hospital | Chiyoko Kono |
| Yamaguchi University Hospital | Keiji Oishi |
| National Hospital Organization Himeji Medical Center/ Clinical Trial Office | Tetsuji Kawamura |
| National Hospital Organization Mie Chuo Medical Center | Hidenori Ibata |
| Saiseikai Kumamoto Hospital | Kazuya Ichikado |
| Kobe City Hospital Organization Kobe City Medical Center West Hospital | Hiromi Tomioka |
| Kobe City Medical Center General Hospital | Keisuke Tomii |
| Fukushima Medical University Hospital | Yoshinori Tanino |
| Tokushima University Hospital | Seidai Sato |
| Nippon Medical School Musashikosugi Hospital | Yoshinobu Saito |
| Sapporo Medical University Hospital | Hirofumi Chiba |
| KKR Toranomon Hospital | Meiyo Tamaoka |
| Jichi Medical University Hospital | Masashi Bando |
| Toho University Omori Medical Center | Susumu Sakamoto |
| National University Corporation Tohoku University Tohoku University Hospital | Naoki Tode |
| Kyushu University Hospital | Isamu Okamoto |
| Hospital of the University of Occupational and Environmental Health, Japan | Kazuhiro Yatera |
| National Hospital Organization Ibrakihigashi National Hospital | Takefumi Saito |
| University of Fukui Hospital | Tamotsu Ishizuka |
| Kyoto University Hospital | Tomohiro Handa |
| Kyorin University Hospital | Haruyuki Ishii |
| Fukuoka University Hospital | Masaki Fujita |
| Nagoya University Hospital | Koji Sakamoto |
| Juntendo University Hospital | Motoyasu Kato |
| Kindai University Hospital | Osamu Nishiyama |
| Tsuboi Hospital | Keishi Sugino |
| Hiroshima Prefectural Hospital | Nobuhisa Ishikawa |
| Hokkaido University Hospital | Hirokazu Kimura |
| Okinawa Prefectural Chubu Hospital | Tomoo Kishaba |
| Tokai University Hospital | Koichiro Asano |
| Saitama Red Cross Hospital | Hidekazu Matsushima |
| Niigata University Medical and Dental Hospital | Yosuke Kimura |

Supplemental Methods

Study design

This study consisted of two phases: the primary assessment phase and the continuous assessment phase. In the primary assessment phase, the treatment period was 26 weeks from the date of the initial dose of study treatment. In the continuous assessment phase, the treatment period was up to 52 weeks from the date of initial dose in the primary assessment phase. For patients who had not completed 26 weeks of treatment at the date of the randomization of the 240^th^ enrolled patient, the treatment period was up to the latest scheduled visit, but was not to exceed 26 weeks after the date of randomization of the 240^th^ patient. For patients randomized after the 241^st^ patient, the treatment period was up to 26 weeks. The study design is summarized in Figure E1.

Key inclusion and exclusion criteria

*Inclusion criteria*

- Is ≥20 years of age at the time of informed consent
- Diagnosed with interstitial lung disease (ILD) by the investigator, and treated with nintedanib or pirfenidone for any of the approved indications for at least 90 days as of Visit 1
- Had fibrosis in >10% of the total lung field, as assessed by central review of chest high-resolution computed tomography (HRCT) images at Visit 1
- Meets the following criteria for progressive disease:

1. Relative decline in percent predicted forced vital capacity (%FVC) of ≥ 5%/year at Visit 1, as calculated below:

| %FVC at basepoint − %FVC at Visit 1 | × | 365 (day) | × 100 ≥5% |
| --- | --- | --- | --- |
| %FVC at basepoint |  | Measurement interval (day) |  |

1. However, when the measurement interval is <365 days, the relative decline in %FVC must be ≥ 5%, as calculated below:

| %FVC at basepoint − %FVC at Visit 1 | × 100 ≥5% |
| --- | --- |
| %FVC at basepoint |  |

1. The basepoint data is defined as the data obtained immediately before or during antifibrotic therapy (nintedanib or pirfenidone) given as of Visit 1, which will be determined by the investigator. The interval between the measurement date of the basepoint data and the measurement at Visit 1 must be ≥ 90 days

- Difference between the FVC at Visit 2 and the FVC at Visit 1 of ≤ +5% or ≤ 150 mL, as calculated below:

| FVC at Visit 2 – FVC at Visit 1 | × 100 ≤5% |
| --- | --- |
| FVC at Visit 1 |  |

Or FVC at Visit 2 – FVC ≤150 mL at Visit 1

- %FVC at Visit 2 of ≥50%
- Percent predicted diffusing capacity for carbon monoxide (%Dlco) [corrected for the haemoglobin (Hb) level at Visit 1] of ≥25%
- Not newly started or discontinued treatment for ILD, other than nintedanib and pirfenidone, within 42 days before randomization
- Larger extent of fibrosis than the extent of emphysematous lesions, as assessed by chest HRCT images at Visit 1 (Central review will be performed, but the final decision will be made by the investigator)

*Exclusion* *criteria*

- Has airflow obstruction (forced expiratory volume in 1 second [FEV_1_]/FVC before bronchodilator use at Visit 2 is < 0·7)
- Diagnosed with severe pulmonary hypertension
- History of acute ILD exacerbation
- Has moderate or severe complications, such as decreased appetite, malaise, diarrhea, nausea, and skin disorder, or moderate or severe adverse reactions to prior treatment for ILD (excluding alopecia/pigmentation)

Procedures

In the previous phase 2a study, the initial pamufetinib dose was 200 mg (E1). In the previous study, patients who received a reduced pamufetinib dose of 100 mg in the 6 weeks after starting treatment showed an FVC trend at 13 and 26 weeks that was consistent with the overall trend (E1). Based on these results, and considering the continuity of administration, the high-dose group for pamufetinib was set at 100 mg and the low-dose group was set at 50 mg in the current study. The initial dose for the control group (nintedanib or pirfenidone) was the same as that prescribed to patients immediately before randomization. Dose reductions and increases in pamufetinib were prohibited during the study period.

Further endpoints

*Time to First Acute Exacerbation*

Time to first acute exacerbation is defined as the period to the date of confirmation of the first acute exacerbation between the date of randomization and Week 52 or the post-observation, whichever comes later. For patients who have not reached the Week 26 or patients who have been randomized from the 241^st^ onwards as of the date of randomization of the 240^th^ patient, time to first acute exacerbation is defined as the period to the date of confirmation of the first acute exacerbation between the date of randomization and Week 26 or the post-observation, whichever comes later. The definitions of the first acute exacerbation event or censoring are provided in following Table. Patients who die without confirmed acute exacerbation will be censored at the date of death. Patients without confirmed acute exacerbation during the period will be censored at the last confirmation date during the period.

**Table: Definitions of First Acute Exacerbation Event or Censoring**

| Inclusion/exclusion of event | Description | Date |
| --- | --- | --- |
| Event | First acute exacerbation | Date of confirmed first acute exacerbation |
| Censoring | Death | Date of death |
|  | Other than the above | Last confirmation date during the period |

Statistical methodology for the primary analysis

For the primary analysis, the rate of decline in FVC at each time point (weeks) was estimated using a restricted maximum likelihood-based linear mixed-effects model. The fixed effects of the linear mixed-effects model included the treatment group, baseline FVC (mL), evaluation time point (weeks) as random effects, and interactions between treatment group and evaluation time point, and between baseline FVC and evaluation time point as fixed effects. Assuming that the degree of the rate of decline in FVC varied among patients, the effect of the difference in patients was included as a random effect for the intercept of the linear mixed-effects model. The degrees of freedom were calculated using the Kenward–Roger method. Based on the results of a blinded review, the primary analysis was conducted by using a linear mixed-effects model incorporating the degree of emphysema (≤10%, >10%) as a covariate.

The predetermined dose-response types were examined using the least-squares mean of the 26-week rate of decline in FVC in each treatment group in the FAS. The following dose-response types (100 mg, 50 mg, and control groups) were investigated: linear (1:0:−1), low-dose saturated (1:1:−2), high-dose saturated (2:−1:−1), and high-dose descending (1:2:−3). A resampling approach was used to adjust for multiplicity while testing the four dose-response types. Based on the contrast test, when the smallest adjusted p-value was <2.5% (one-sided significance level), the dose-response was determined to have the smallest adjusted p-value. In the resampling method, resampling was performed one million times, and the adjusted p-value was calculated.

In the primary analysis, the mechanism of missing data was assumed to random; therefore, missing FVC data were not imputed. All available FVC data up to week 26 before the start of the subsequent treatment were included in the primary analysis.

Supplemental Results

Supplemental Tables

**Table E1. Summary of Clinical Interstitial Lung Disease Diagnoses at Baseline in the Full Analysis Set**

| ***n* (%)** | **Pamufetinib 100 mg (*n* = 78)** | **Pamufetinib 50 mg (*n* = 83)** | **Control (*n* = 80)** | **Total (*N* = 241)** |
| --- | --- | --- | --- | --- |
| Diagnosis of ILD (Category 1) |  |  |  |  |
| IPF | 57 (73.1) | 61 (73.5) | 53 (66.3) | 171 (71.0) |
| Idiopathic non-specific interstitial pneumonia | 2 (2.6) | 3 (3.6) | 2 (2.5) | 7 (2.9) |
| Unclassifiable idiopathic interstitial pneumonia | 10 (12.8) | 7 (8.4) | 10 (12.5) | 27 (11.2) |
| Hypersensitivity pneumonitis | 5 (6.4) | 8 (9.6) | 3 (3.8) | 16 (6.6) |
| Rheumatoid arthritis-associated ILD | 1 (1.3) | 1 (1.2) | 3 (3.8) | 5 (2.1) |
| Mixed connective tissue disease | 0 | 0 | 0 | 0 |
| Systemic sclerosis-associated ILD | 1 (1.3) | 0 | 4 (5.0) | 5 (2.1) |
| Exposure-related ILD | 0 | 1 (1.2) | 0 | 1 (0.4) |
| Sarcoidosis | 0 | 0 | 0 | 0 |
| Other fibrosing ILD | 2 (2.6) | 2 (2.4) | 5 (6.3) | 9 (3.7) |
| Diagnosis of ILD (Category 2) |  |  |  |  |
| IPF | 57 (73.1) | 61 (73.5) | 53 (66.3) | 171 (71.0) |
| Non-IPF | 21 (26.9) | 22 (26.5) | 27 (33.8) | 70 (29.0) |

*Definition of abbreviations*: ILD = interstitial lung disease, IPF = idiopathic pulmonary fibrosis

**Table E2. Summary of Demographics and Other Baseline Characteristics in the Idiopathic Pulmonary Fibrosis and Non-Idiopathic Pulmonary Fibrosis Populations**

|  | **IPF population** | | | **Non-IPF population** | | |
| --- | --- | --- | --- | --- | --- | --- |
|  | **Pamufetinib 100 mg (*n* = 57)** | **Pamufetinib 50 mg (*n* = 61)** | **Control (*n* = 53)** | **Pamufetinib 100 mg (*n =* 21)** | **Pamufetinib 50 mg (*n* = 22)** | **Control (*n* = 27)** |
| Male sex, *n* (%) | 51 (89.5) | 52 (85.2) | 44 (83.0) | 20 (95.2) | 16 (72.7) | 20 (74.1) |
| Age, years, mean ± SD | 69.9 ± 8.4 | 71.3 ± 6.7 | 73.7 ± 5.7 | 66.3 ± 12.2 | 67.6 ± 9.0 | 66.3 ± 10.3 |
| Former or current smoker, *n* (%) | 45 (79.0) | 50 (81.9) | 44 (83.0) | 18 (85.7) | 15 (68.2) | 20 (74.1) |
| Diagnosis of IPF, *n* (%) | 57 (100.0) | 61 (100.0) | 53 (100.0) | - | - | - |
| Duration of ILD from the initial diagnosis, years, mean ± SD | 4.28 ± 2.61 | 4.73 ± 3.17 | 3.73 ± 2.69 | 3.18 ± 2.66 | 4.80 ± 3.66 | 4.06 ± 2.79 |
| Duration of prior nintedanib and/or pirfenidone, years, mean ± SD | 2.37 ± 1.85 | 2.03 ± 1.61 | 2.06 ± 1.37 | 1.38 ± 0.68 | 1.66 ± 1.14 | 1.80 ± 1.30 |
| Prior antifibrotic treatment, *n* (%) |  |  |  |  |  |  |
| Nintedanib | 37 (64.9) | 44 (72.1) | 34 (64.2) | 20 (95.2) | 20 (90.9) | 26 (96.3) |
| Pirfenidone | 14 (24.6) | 11 (18.0) | 12 (22.6) | 0 | 1 (4.5) | 0 |
| Both | 6 (10.5) | 6 (9.8) | 7 (13.2) | 1 (4.8) | 1 (4.5) | 1 (3.7) |
| FVC |  |  |  |  |  |  |
| Value, mL, mean ± SD | 2412.1 ± 617.0 | 2294.3 ± 489.7 | 2244.5 ± 573.4 | 2357.5 ± 365.3 | 2179.1 ± 639.2 | 2226.2 ± 571.5 |
| % predicted, mean ± SD | 75.8 ± 16.2 | 75.0 ± 12.4 | 75.9 ± 15.6 | 68.3 ± 11.6 | 71.1 ± 13.9 | 70.2 ± 11.8 |
| Decline in %FVC, *n* (%) |  |  |  |  |  |  |
| ≥5% and <10% per year | 29 (50.9) | 31 (50.8) | 27 (50.9) | 10 (47.6) | 11 (50.0) | 15 (55.6) |
| ≥10% per year | 28 (49.1) | 30 (49.2) | 26 (49.1) | 11 (52.4) | 11 (50.0) | 12 (44.4) |
| Dlco |  |  |  |  |  |  |
| Value, mL/min/mmHg, mean ± SD | 8.8 ± 2.9 | 9.2 ± 3.0 | 8.1 ± 2.7 | 9.7 ± 3.1 | 9.5 ± 3.7 | 9.0 ± 3.2 |
| % predicted, mean ± SD | 52.6 ± 14.1 | 56.1 ± 15.8 | 53.2 ± 16.6 | 56.0 ± 18.0 | 59.4 ± 22.3 | 51.2 ± 17.5 |

*Definition of abbreviations*: Dlco = diffusing capacity of the lungs for carbon monoxide, FVC =forced vital capacity, %FVC = percent predicted forced vital capacity, ILD = interstitial lung disease, IPF = idiopathic pulmonary fibrosis

**Table E3. Dose-Response Statistical Analysis Results in the Full Analysis Set**

| **Dose Patterns** | **Linear** | **Low-dose saturated** | **High-dose saturated** | **High-dose descending** |
| --- | --- | --- | --- | --- |
| P-Value | 0.9999 | 0.9990 | 0.9998 | 0.9960 |

**Table E4. First Acute Exacerbations of Interstitial Lung Disease in the Full Analysis Set**

|  | **Pamufetinib 100 mg (*n* = 78)** | **Pamufetinib 50 mg (*n* = 83)** | **Control (*n* = 80)** |
| --- | --- | --- | --- |
| Acute exacerbations (events), *n* (%) |  |  |  |
| At week 26 | 10 (12.8) | 8 (9.6) | 3 (3.8) |
| At week 52 | 15 (19.2) | 12 (14.5) | 7 (8.8) |
| Patients at risk for acute exacerbation, *n* (%) |  |  |  |
| At week 26 | 55 (70.5) | 66 (79.5) | 67 (83.8) |
| At week 52 | 27 (34.6) | 35 (42.2) | 39 (48.8) |
| Time to first acute exacerbation, days, median (95% CI) | NR (372.00, NR) | NR (378.00, NR) | NR |
| Restricted mean time to first acute exacerbation, days, median (95% CI) |  |  |  |
| At week 26 | 168.57 (159.44, 177.71) | 174.38 (167.28, 181.49) | 180.47 (176.33, 184.61) |
| At week 52 | 318.41 (293.86, 342.96) | 333.80 (314.71, 352.89) | 346.82 (332.50, 361.14) |
| Event-free rate, % (95% CI) |  |  |  |
| At week 26 | 85.8 (75.2, 92.1) | 89.8 (80.7, 94.8) | 96.1 (88.3, 98.7) |
| At week 52 | 75.8 (62.4, 84.9) | 83.2 (72.1, 90.2) | 89.6 (79.3, 94.9) |
| P-value vs control* | 0.0188 | 0.1798 | – |
| HR (95% CI) vs control | 2.79 (1.15, 6.78) | 1.89 (0.75, 4.73) | – |

*Definition of abbreviations*: CI = confidence interval, HR = hazard ratio, ILD = interstitial lung disease, NR = not reached

*Measured using a log-rank test

**Table E5. Overall Survival in the Full Analysis Set**

|  | **Pamufetinib 100 mg (*n* = 78)** | **Pamufetinib 50 mg (*n* = 83)** | **Control (*n* = 80)** |
| --- | --- | --- | --- |
| OS at week 26 |  |  |  |
| Deaths , *n* (%) | 3 (3.8) | 5 (6.0) | 2 (2.5) |
| At risk, *n* (%) | 59 (75.6) | 68 (81.9) | 69 (86.3) |
| Restricted mean OS, days (95% CI) | 180.16 (175.54, 184.78) | 178.63 (174.18, 183.08) | 182.52 (181.51, 183.53) |
| Event-free rate, % (95% CI) | 95.6 (86.8, 98.5) | 93.5 (85.0, 97.2) | 97.3 (89.5, 99.3) |
| OS at week 52 |  |  |  |
| Deaths, *n* (%) | 3 (3.8) | 11 (13.3) | 8 (10.0) |
| At risk, *n* (%) | 32 (41.0) | 37 (44.6) | 41 (51.3) |
| Restricted mean OS, days (95% CI) | 354.06 (339.10, 369.03) | 341.76 (325.92, 357.60) | 349.83 (338.58, 361.08) |
| Event-free rate, % (95% CI) | 95.6 (86.8, 98.5) | 83.5 (72.0, 90.6) | 87.7 (76.9, 93.7) |
| P-value vs control* | 0.2031 | 0.4677 | – |
| HR (95% CI) vs control | 0.44 (0.12, 1.64) | 1.40 (0.56, 3.48) | – |

*Definition of abbreviations*: CI = confidence interval, HR = hazard ratio, ILD = interstitial lung disease, OS = overall survival

*Measured using a log-rank test

**Table E6. Adverse Events up to Week 26 in the Safety Population by Subgroup**

| ***n* (%)** | **Pamufetinib 100 mg** | | **Pamufetinib 50 mg** | | **Control** | |
| --- | --- | --- | --- | --- | --- | --- |
|  | **IPF (*n =* 58)** | **Non-IPF (*n =* 21)** | **IPF (*n =* 62)** | **Non-IPF (*n =* 22)** | **IPF (*n =* 53)** | **Non-IPF (*n =* 27)** |
| Any AE* | 54 (93.1) | 19 (90.5) | 53 (85.5) | 18 (81.8) | 46 (86.8) | 25 (92.6) |
| Most frequent AEs† |  |  |  |  |  |  |
| Rash | 14 (24.1) | 9 (42.9) | 9 (14.5) | 3 (13.6) | 6 (11.3) | 2 (7.4) |
| Diarrhea | 5 (8.6) | 0 | 3 (4.8) | 1 (4.5) | 11 (20.8) | 5 (18.5) |
| Pyrexia | 5 (8.6) | 3 (14.3) | 5 (8.1) | 0 | 5 (9.4) | 3 (11.1) |
| COVID-19 | 4 (6.9) | 1 (4.8) | 5 (8.1) | 4 (18.2) | 3 (5.7) | 4 (14.8) |
| ILD‡ | 5 (8.6) | 3 (14.3) | 6 (9.7) | 2 (9.1) | 2 (3.8) | 1 (3.7) |
| Constipation | 4 (6.9) | 4 (19.0) | 4 (6.5) | 1 (4.5) | 3 (5.7) | 0 |
| Decreased appetite | 2 (3.4) | 0 | 1 (1.6) | 0 | 6 (11.3) | 5 (18.5) |
| Severe AEs§ | 13 (22.4) | 7 (33.3) | 11 (17.7) | 4 (18.2) | 8 (15.1) | 3 (11.1) |
| Serious AEsll | 15 (25.9) | 7 (33.3) | 12 (19.4) | 5 (22.7) | 8 (15.1) | 4 (14.8) |
| Fatal AEs | 1 (1.7) | 2 (9.5) | 4 (6.5) | 1 (4.5) | 2 (3.8) | 0 |
| AEs leading to treatment discontinuation | 6 (10.3) | 4 (19.0) | 9 (14.5) | 1 (4.5) | 0 | 0 |

*Definition of abbreviations*: AE(s) = adverse event(s), COVID-19 = coronavirus disease 2019, CTCAE = Common Terminology Criteria for Adverse Events; ILD = interstitial lung disease, IPF = idiopathic pulmonary fibrosis; MedDRA = Medical Dictionary for Regulatory Activities

*AEs were coded using MedDRA, version 27.0

†The most frequent AEs were defined as those with an incidence >10% in any study group

‡ILD was reported as an AE, encompassing both acute ILD exacerbations and newly diagnosed ILD

§A severe AE was defined as an AE of Grade ≥3 in the CTCAE, version 5.0

llA serious AE was defined as any AE that resulted in death, was life-threatening, required inpatient hospitalization or prolongation of existing hospitalization to treat the AE, resulted in persistent or significant disability/incapacity, was a congenital anomaly/birth defect, or was deemed serious for any other reason

**Table E7. Adverse Events Occurring in >5% of Patients in any Study Group up to Week 26 in the Safety Population**

| **AEs, *n* (%)***  **System Organ Class**  **Preferred Term** | **Pamufetinib 100 mg (*n* = 79)** | **Pamufetinib 50 mg (*n* = 84)** | **Control (*n* = 80)** |
| --- | --- | --- | --- |
| Any AE | 73 (92.4) | 71 (84.5) | 71 (88.8) |
| Eye disorders | 12 (15.2) | 11 (13.1) | 5 (6.3) |
| Eyelid edema | 7 (8.9) | 7 (8.3) | 1 (1.3) |
| Gastrointestinal disorders | 24 (30.4) | 18 (21.4) | 29 (36.3) |
| Constipation | 8 (10.1) | 5 (6.0) | 3 (3.8) |
| Diarrhea | 5 (6.3) | 4 (4.8) | 16 (20.0) |
| General disorders and administration site conditions | 20 (25.3) | 13 (15.5) | 15 (18.8) |
| Pyrexia | 8 (10.1) | 5 (6.0) | 8 (10.0) |
| Edema peripheral | 4 (5.1) | 2 (2.4) | 2 (2.5) |
| Malaise | 4 (5.1) | 2 (2.4) | 2 (2.5) |
| Face edema | 4 (5.1) | 2 (2.4) | 0 |
| Infections and infestations | 29 (36.7) | 29 (34.5) | 26 (32.5) |
| Pneumonia | 6 (7.6) | 4 (4.8) | 3 (3.8) |
| COVID-19 | 5 (6.3) | 9 (10.7) | 7 (8.8) |
| Nasopharyngitis | 3 (3.8) | 7 (8.3) | 7 (8.8) |
| Investigations | 22 (27.8) | 11 (13.1) | 14 (17.5) |
| Blood creatine phosphokinase increased | 4 (5.1) | 3 (3.6) | 0 |
| Aspartate aminotransferase increased | 4 (5.1) | 0 | 1 (1.3) |
| Weight decreased | 1 (1.3) | 0 | 6 (7.5) |
| Metabolism and nutrition disorders | 12 (15.2) | 8 (9.5) | 13 (16.3) |
| Hyperglycemia | 4 (5.1) | 0 | 1 (1.3) |
| Decreased appetite | 2 (2.5) | 1 (1.2) | 11 (13.8) |
| Hypokalemia | 0 | 5 (6.0) | 0 |
| Nervous system disorders | 12 (15.2) | 11 (13.1) | 12 (15.0) |
| Dysgeusia | 4 (5.1) | 0 | 0 |
| Headache | 1 (1.3) | 5 (6.0) | 5 (6.3) |
| Psychiatric disorders | 7 (8.9) | 7 (8.3) | 2 (2.5) |
| Insomnia | 7 (8.9) | 7 (8.3) | 2 (2.5) |
| Respiratory, thoracic and mediastinal disorders | 23 (29.1) | 24 (28.6) | 24 (30.0) |
| ILD | 8 (10.1) | 8 (9.5) | 3 (3.8) |
| Dyspnea | 7 (8.9) | 5 (6.0) | 7 (8.8) |
| Cough | 4 (5.1) | 3 (3.6) | 4 (5.0) |
| Pneumothorax | 4 (5.1) | 3 (3.6) | 2 (2.5) |
| Skin and subcutaneous tissue disorders | 40 (50.6) | 33 (39.3) | 21 (26.3) |
| Rash | 23 (29.1) | 12 (14.3) | 8 (10.0) |
| Eczema | 5 (6.3) | 3 (3.6) | 3 (3.8) |
| Erythema | 4 (5.1) | 2 (2.4) | 1 (1.3) |
| Pruritus | 3 (3.8) | 7 (8.3) | 4 (5.0) |

*Definition of abbreviations*: AE(s) = adverse event(s), COVID-19 = coronavirus disease 2019, ILD = interstitial lung disease, MedDRA = Medical Dictionary for Regulatory Activities

*AEs were coded using MedDRA, version 27.0

**Table E8. Treatment-Related Adverse Event Occurring in >5% of Patients in any Study Group up to Week 26 in the Safety Population**

| **Treatment-related AEs, *n* (%)***  **System Organ Class**  **Preferred Term** | **Pamufetinib 100 mg (*n* = 79)** | **Pamufetinib 50 mg (*n* = 84)** | **Control (*n* = 80)** |
| --- | --- | --- | --- |
| Any treatment-related AE | 54 (68.4) | 40 (47.6) | 35 (43.8) |
| Eye disorders | 10 (12.7) | 6 (7.1) | 0 |
| Eyelid edema | 7 (8.9) | 6 (7.1) | 0 |
| Gastrointestinal disorders | 10 (12.7) | 6 (7.1) | 15 (18.8) |
| Diarrhea | 4 (5.1) | 3 (3.6) | 13 (16.3) |
| General disorders and administration site conditions | 11 (13.9) | 8 (9.5) | 2 (2.5) |
| Malaise | 4 (5.1) | 2 (2.4) | 1 (1.3) |
| Edema peripheral | 4 (5.1) | 1 (1.2) | 1 (1.3) |
| Investigations | 19 (24.1) | 9 (10.7) | 6 (7.5) |
| Blood creatine phosphokinase increased | 4 (5.1) | 3 (3.6) | 0 |
| Metabolism and nutrition disorders | 3 (3.8) | 2 (2.4) | 8 (10.0) |
| Decreased appetite | 2 (2.5) | 0 | 8 (10.0) |
| Skin and subcutaneous tissue disorders | 32 (40.5) | 19 (22.6) | 11 (13.8) |
| Rash | 19 (24.1) | 10 (11.9) | 6 (7.5) |
| Erythema | 4 (5.1) | 2 (2.4) | 1 (1.3) |
| Eczema | 4 (5.1) | 1 (1.2) | 1 (1.3) |

*Definition of abbreviations*: AE(s) = adverse event(s), MedDRA = Medical Dictionary for Regulatory Activities

*AEs were coded using MedDRA version 27.0

**Table E9. Adverse Events Leading to Death Over the Entire Study Period in the Safety Population**

| **Treatment** | **Sex** | **AE*** | **Relationship** |
| --- | --- | --- | --- |
| Pamufetinib 100 mg | Male | ILD | Not related |
| Pamufetinib 100 mg | Female | Chronic respiratory failure/ILD | Not related |
| Pamufetinib 100 mg | Male | ILD | Not related |
| Pamufetinib 100 mg | Male | Pneumothorax | Not related |
| Pamufetinib 100mg | Female | Subarachnoid hemorrhage | Not related |
| Pamufetinib 50 mg | Male | ILD | Not related |
| Pamufetinib 50 mg | Male | COVID-19 | Not related |
| Pamufetinib 50 mg | Female | Respiratory disorder/Right ventricular dysfunction | Not related |
| Pamufetinib 50 mg | Male | ILD | Not related |
| Pamufetinib 50 mg | Male | ILD | Not related |
| Pamufetinib 50 mg | Male | ILD | Related |
| Pamufetinib 50 mg | Male | ILD | Not related |
| Control | Male | Acute respiratory distress syndrome | Not related |
| Control | Male | Pneumothorax | Not related |
| Control | Male | Dyspnea | Not related |
| Control | Male | Cardio-respiratory arrest | Not related |

*Definition of abbreviations*: AE(s) = adverse event(s), ILD = interstitial lung disease, MedDRA = Medical Dictionary for Regulatory Activities

*AEs were coded using MedDRA, version 27.0

Supplemental Figures

**Figure E1.** Study design. FVC = forced vital capacity; NTD = nintedanib; PFD = pirfenidone; R = randomization; w = weeks.


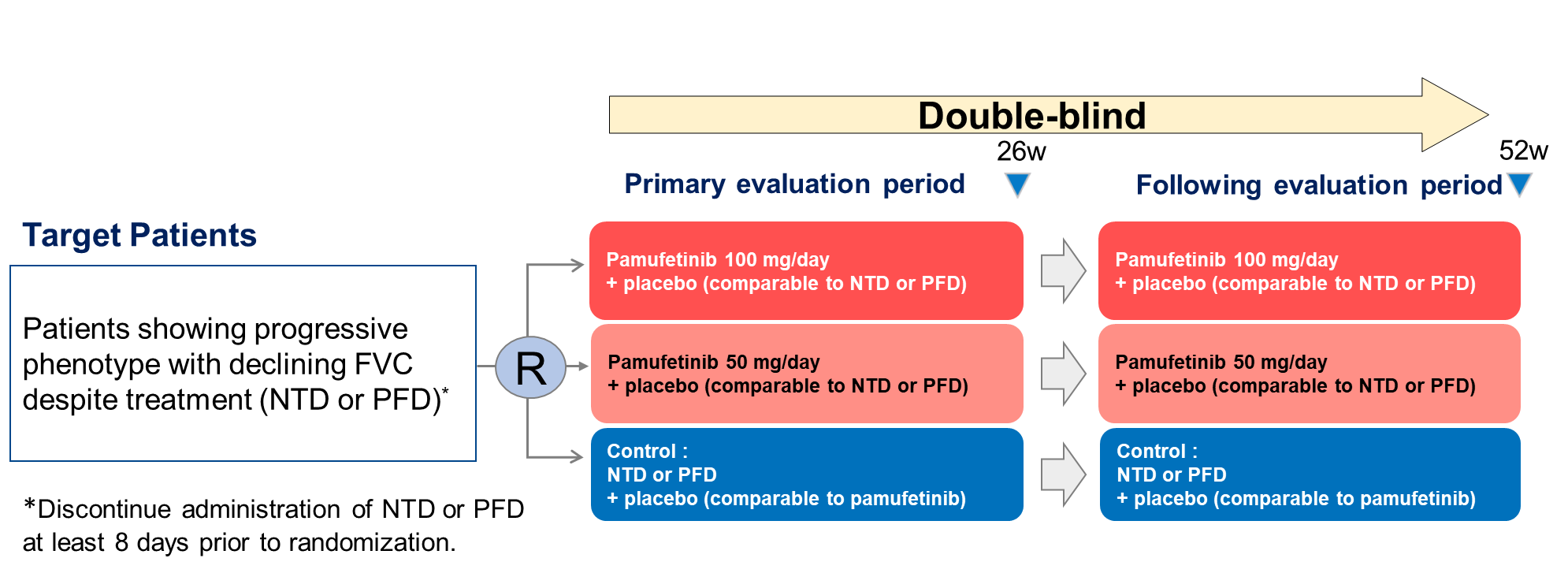


**Figure E2.** Change in forced vital capacity from baseline over time in patients with or without study drug-related skin disorders* in the full analysis set (a) in the IPF population and (b) in the non-IPF population. Error bars indicate standard error. *Skin disorders were classified using MedDRA, version 27.0, and included any terms listed in the System Organ Class “skin and subcutaneous tissue disorders” category. FVC = forced vital capacity, IPF = idiopathic pulmonary fibrosis


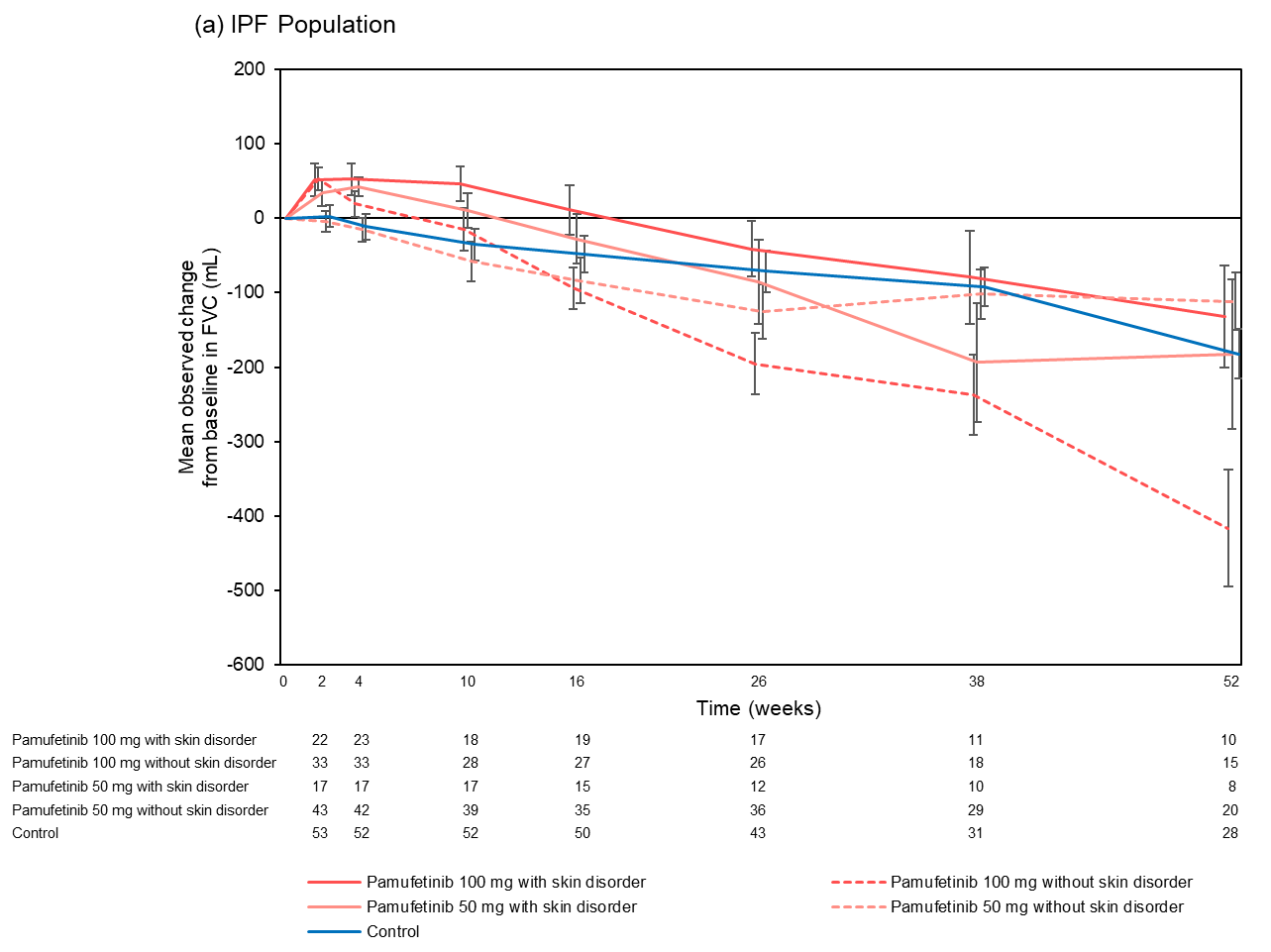


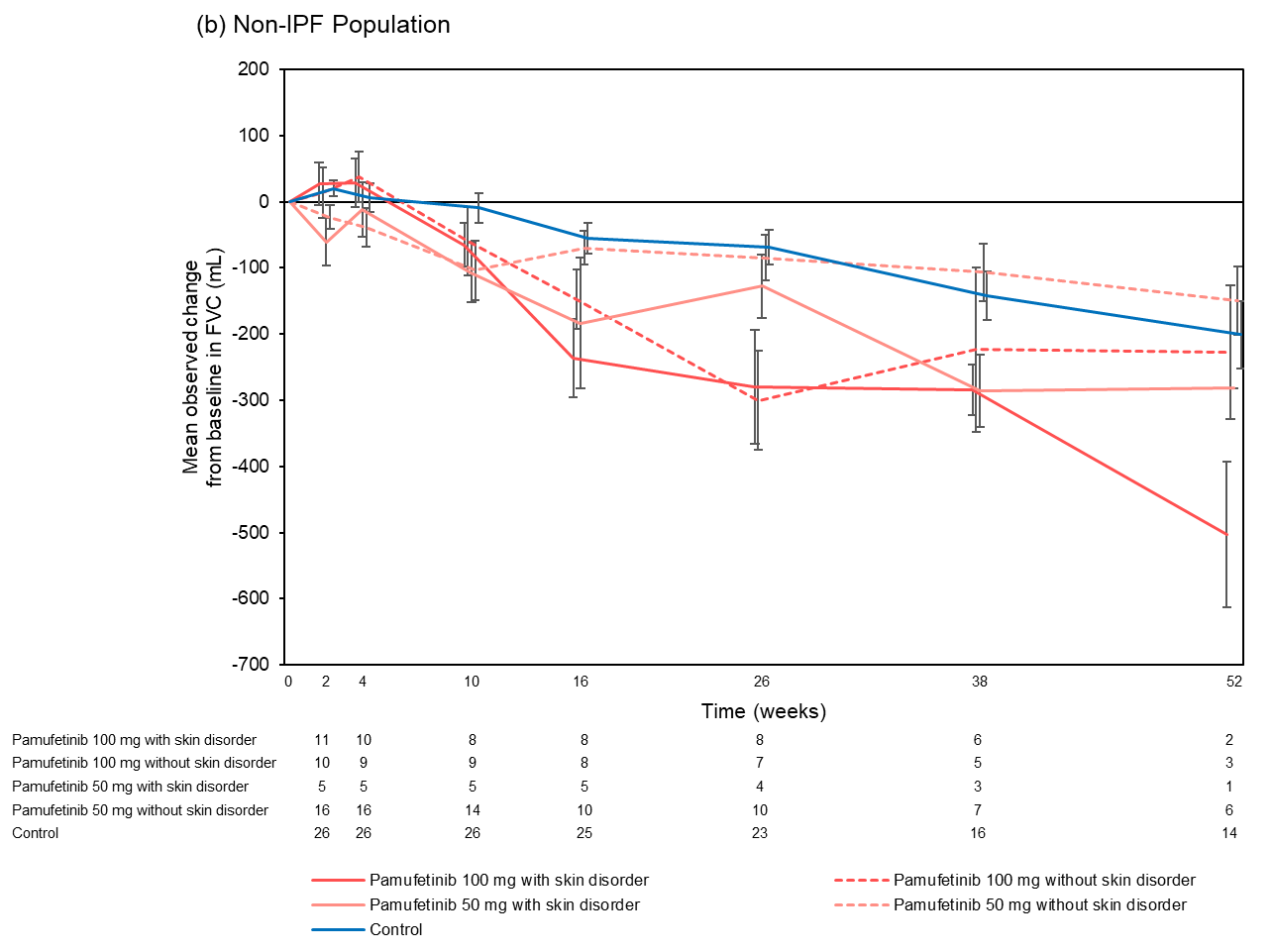


**Figure E3.** Time to first acute exacerbation in the full analysis set (a) in the overall population, (b) in the IPF population, and (c) in the non-IPF population. Lines indicate censored events. FVC = forced vital capacity, IPF = idiopathic pulmonary fibrosis


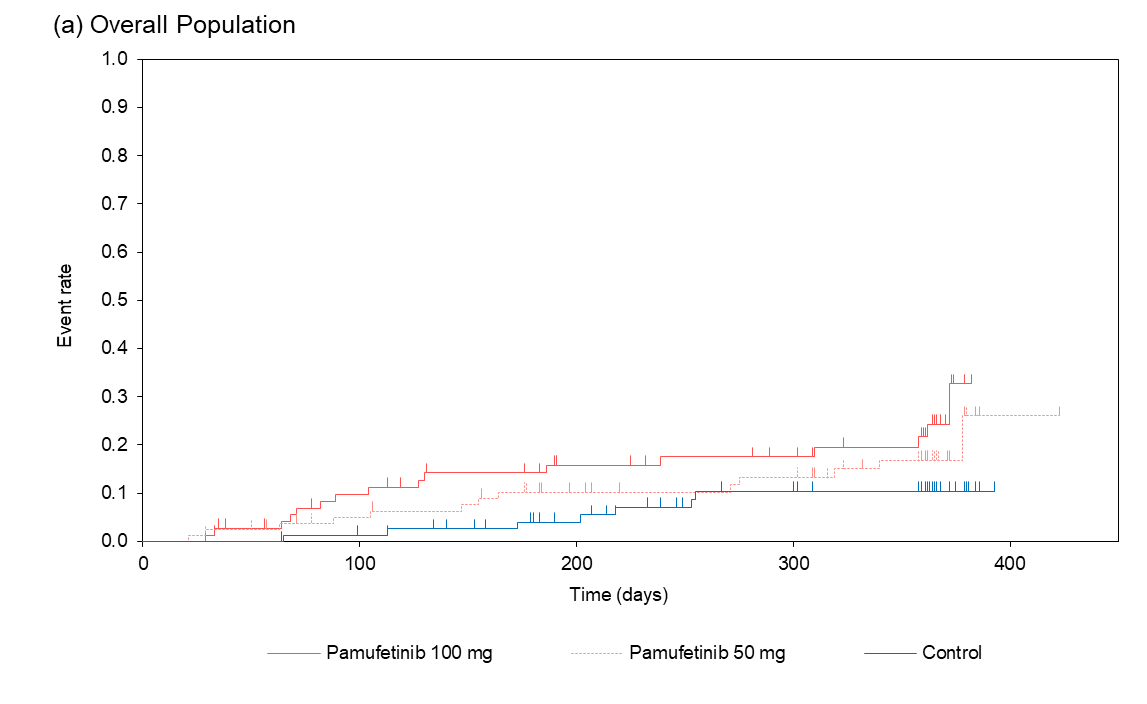


***
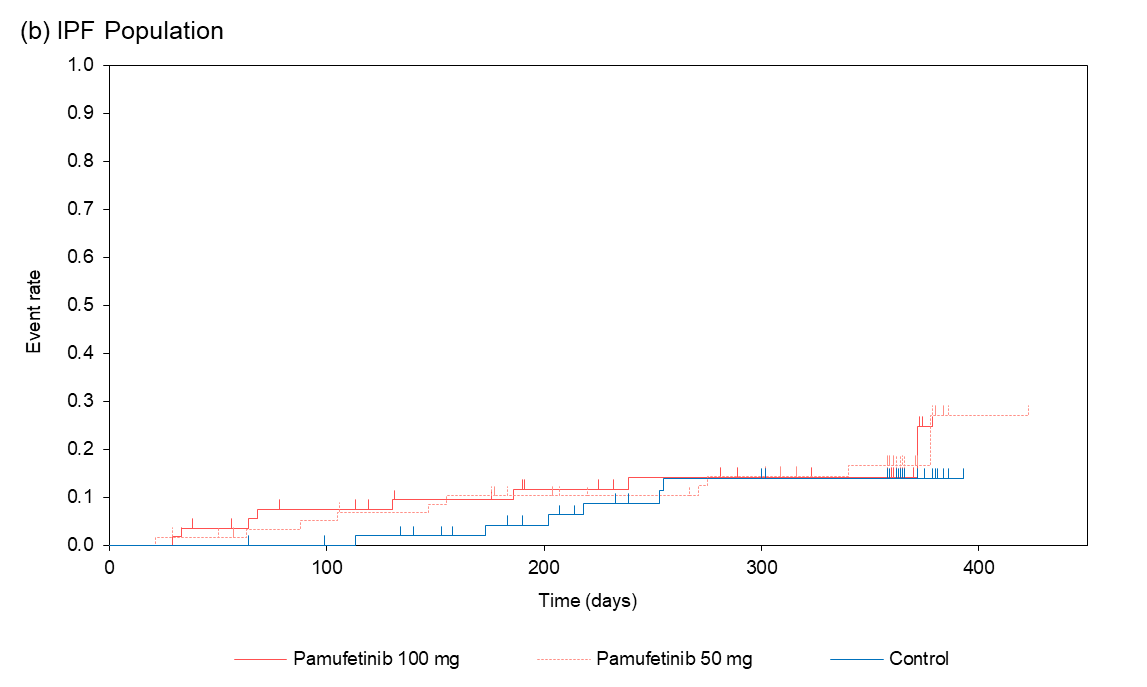
***

***
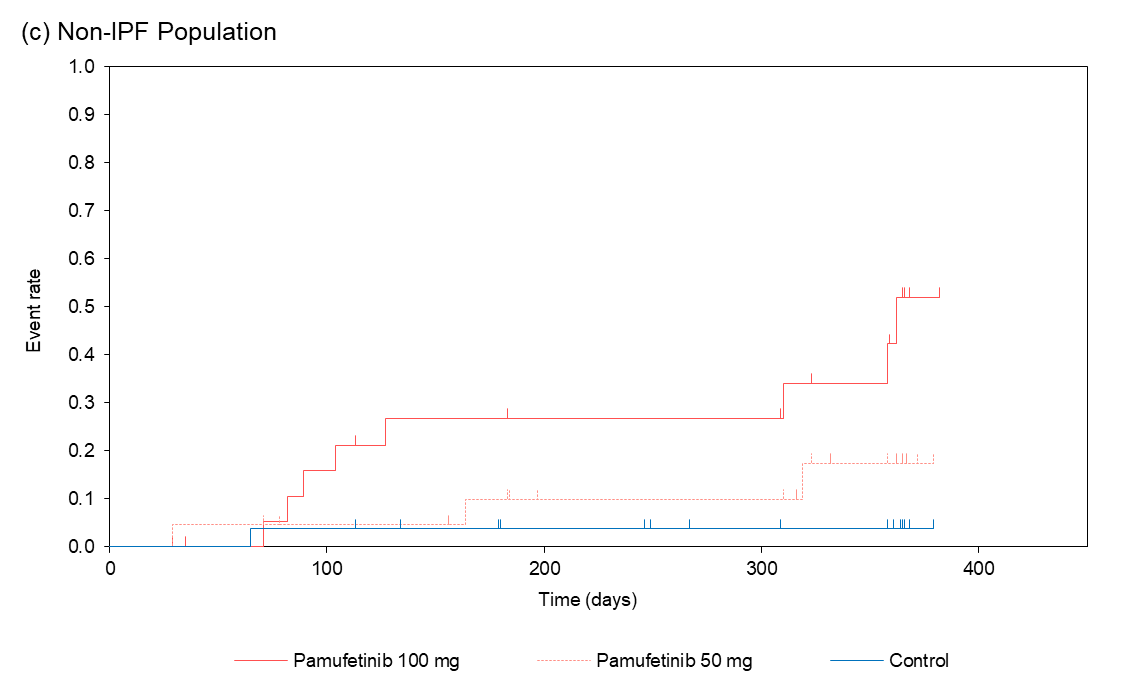
***

**Figure E4.** Kaplan-Meier curve of overall survival in the full analysis set. Lines indicate censored events.


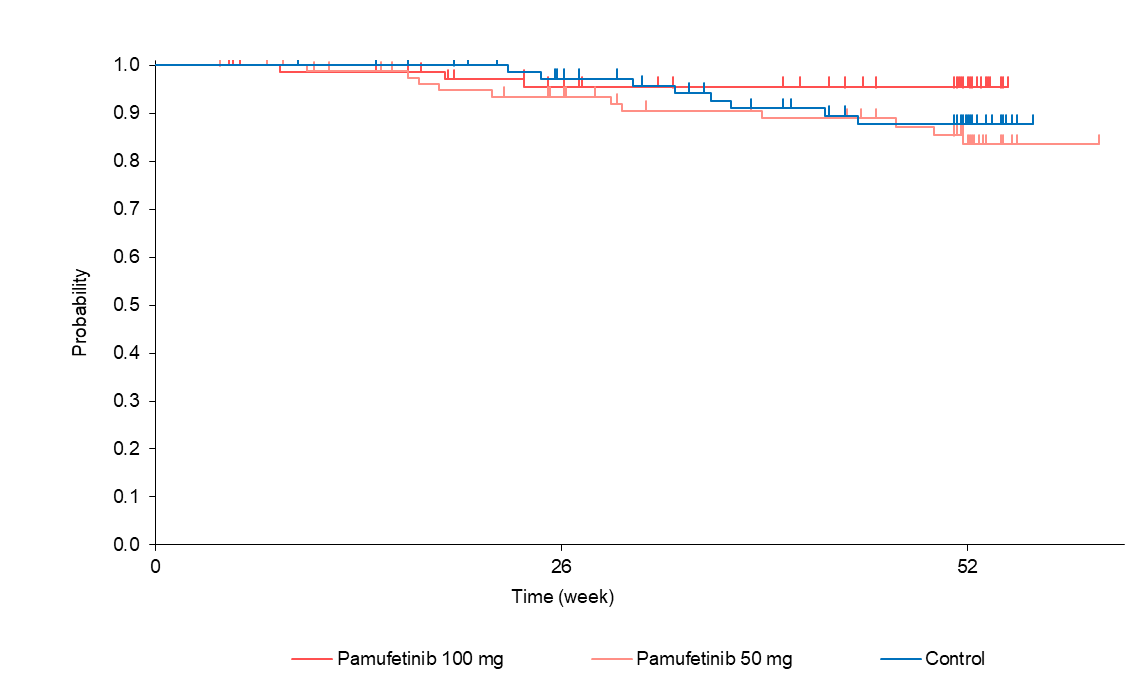


**Figure E5.** Change in St. George's Respiratory Questionnaire scores over time in the full analysis set. Error bars indicate standard error. A linear mixed effect model was conducted that included treatment group, baseline value, assessment time, and interaction between treatment group as random effects, and interaction between baseline value and assessment time as fixed effects. SGRQ = St. George's Respiratory Questionnaire


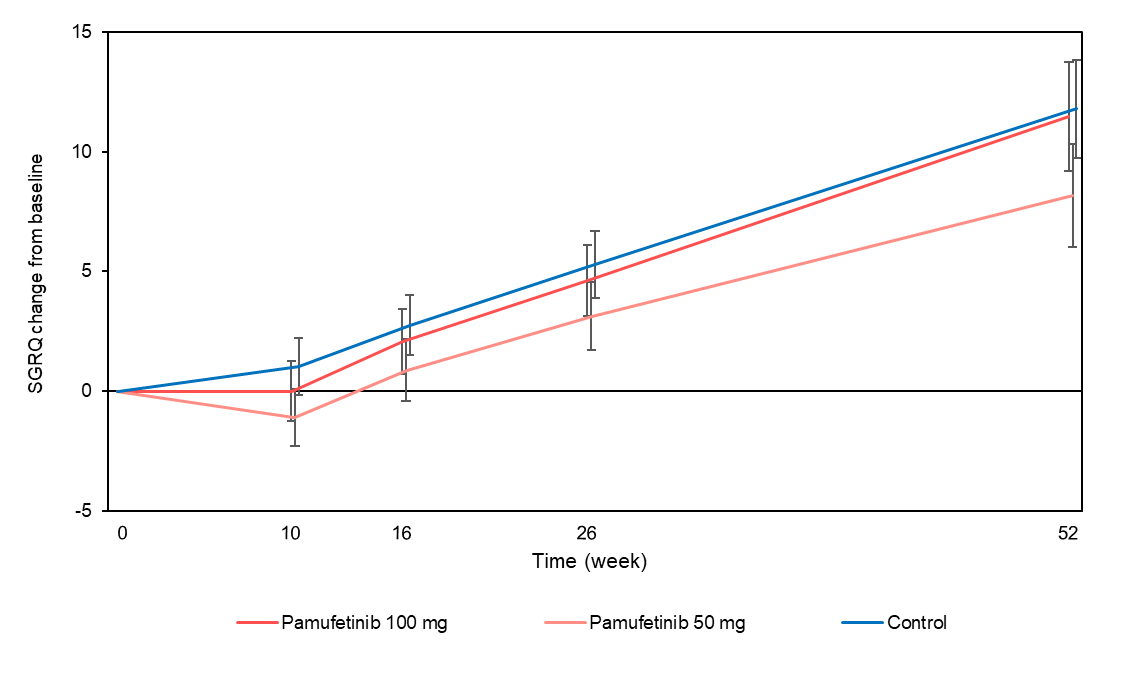


# References

E1. Nishioka Y, Homma S, Ogura T, Sato S, Arai N, Tomii K, et al. Exploratory phase 2 study of the novel oral multi-kinase inhibitor TAS-115 in patients with idiopathic pulmonary fibrosis. *Respir Investig* 2023;61:498-507.
